# Clinical predictive value of the CRP-albumin-lymphocyte index for prognosis of critically ill patients with sepsis in intensive care unit: a retrospective single-center observational study

**DOI:** 10.3389/fpubh.2024.1395134

**Published:** 2024-05-22

**Authors:** Jinhui Zhang, Qun Zhao, Shuo Liu, Nana Yuan, Zhenkui Hu

**Affiliations:** Department of Critical Care Medicine, The Affiliated Hospital, Jiangsu University, Zhenjiang, Jiangsu, China

**Keywords:** sepsis, CRP-albumin-lymphocyte index, inflammation, nutrition, immune, prognosis

## Abstract

**Background:**

Sepsis is a complex syndrome characterized by physiological, pathological, and biochemical abnormalities caused by infection. Its development is influenced by factors such as inflammation, nutrition, and immune status. Therefore, we combined C-reactive protein (CRP), albumin, and lymphocyte, which could reflect above status, to be the CRP-albumin-lymphocyte (CALLY) index, and investigated its association with clinical prognosis of critically ill patients with sepsis.

**Methods:**

This retrospective observational study enrolled critically ill patients with sepsis who had an initial CRP, albumin, and lymphocyte data on the first day of ICU admission. All data were obtained from the Affiliated Hospital of Jiangsu University. The patients were divided into quartiles (Q1–Q4) based on their CALLY index. The outcomes included 30-/60-day mortality and acute kidney injury (AKI) occurrence. The association between the CALLY index and these clinical outcomes in critically ill patients with sepsis was evaluated using Cox proportional hazards and logistic regression analysis.

**Results:**

A total of 1,123 patients (63.0% male) were included in the study. The 30-day and 60-day mortality rates were found to be 28.1 and 33.4%, respectively, while the incidence of AKI was 45.6%. Kaplan–Meier analysis revealed a significant association between higher CALLY index and lower risk of 30-day and 60-day mortality (log-rank *p* < 0.001). Multivariate Cox proportional hazards analysis indicated that the CALLY index was independently associated with 30-day mortality [HR (95%CI): 0.965 (0.935–0.997); *p* = 0.030] and 60-day mortality [HR (95%CI): 0.969 (0.941–0.997); *p* = 0.032]. Additionally, the multivariate logistic regression model showed that the CALLY index served as an independent risk predictor for AKI occurrence [OR (95%CI): 0.982 (0.962–0.998); *p* = 0.033].

**Conclusion:**

The findings of this study indicated a significant association between the CALLY index and both 30-day and 60-day mortality, as well as the occurrence of AKI, in critically ill patients with sepsis. These findings suggested that the CALLY index may be a valuable tool in identifying sepsis patients who were at high risk for unfavorable outcomes.

## Introduction

Sepsis was a condition characterized by a systemic inflammatory response syndrome triggered by the invasion of pathogenic microorganisms, particularly bacteria, into the body (1). In severe cases, sepsis can progress to septic shock, disseminated intravascular coagulation, and multiple organ dysfunction syndrome. These complications contributed significantly to the mortality rate among critically ill patients in the intensive care unit (ICU) (2). Despite advancements in sepsis guidelines and more standardized treatments, the mortality rate associated with this condition remains alarmingly high (3). Globally, sepsis posed a substantial burden, with an estimated 31.5 million cases and 5.3 million deaths occurring annually. It was responsible for approximately 35% of all hospital deaths, with an overall mortality rate ranging between 20 and 30% (4–6). To improve clinical outcomes, early recognition, assessment, and intervention were crucial in the management of sepsis (7). Previous studies had identified several prognostic factors such as oncostatin M (OSM), apoptosis inhibitor of macrophage (AIM/CD5L), interleukin-26 (IL-26), interleukin-17D (IL-17D), and growth differentiation factor-15 (GDF-15) (8–12). However, these factors had limitations and might not provide sufficient prognostic information to guide survival prediction or facilitate the selection of effective treatment strategies. To enhance patient outcomes in sepsis, it was imperative for researchers to identify more accurate predictive factors that can inform therapy decisions.

The pathogenesis and etiology of sepsis were complex and currently lack a definitive consensus. Previous research had demonstrated that the development of sepsis was influenced by various factors, including inflammation levels, nutritional status, and immune function (13–17). The systemic inflammatory response served as a crucial indicator of sepsis progression, with patients exhibiting higher levels of inflammation facing a greater risk of mortality compared to those with lower levels of inflammation (18). Moreover, nutritional status played a significant role in the prognosis of sepsis patients, as studies had shown that undernourished individuals tend to have poorer overall survival (19–21). Furthermore, robust immune function acted as the primary defense against sepsis progression, with patients having impaired immune function experiencing more severe outcomes (22–24). Considering these theories and findings, it was believed that an indicator encompassing inflammation, nutritional status, and immune function could provide more comprehensive prognostic information for sepsis patients.

Clinical and previous studies had frequently utilized hematological indicators to assess the inflammation level, nutritional status, and immune function in patients with sepsis (24–26). Specifically, several key indicators had been identified: C-reactive protein (CRP): As an acute-phase response protein regulated by interleukin-6, CRP served as a clinically recognized marker of the inflammatory response (27). Research by Cui et al. (26) had established a close relationship between CRP levels and poor prognosis in sepsis patients. Albumin: synthesized in the liver, serum albumin was commonly used as an indicator of both nutritional status and disease severity (28). It also reflected the inflammatory response, and hypoalbuminemia had strong predictive value for unfavorable outcomes in sepsis patients (29). Lymphocyte: a traditional biomarker widely employed to evaluate immunocompetence, lymphocyte count was highly relevant to sepsis prognosis (30, 31). Lymphocytopenia was considered a predictor of impaired immunity and unfavorable outcomes in sepsis patients (32). In addition, researchers had recently developed a composite parameter called the CRP-albumin-lymphocyte (CALLY) index, which comprised C-reactive protein content, serum albumin levels, and lymphocyte count (33). This index incorporated indicators of inflammation level, nutritional status, and immune function. Given its ease of obtainment in clinical settings, the CALLY index had garnered increased attention. Previous studies had demonstrated its potential value in predicting disease outcomes in various cancers, such as hepatocellular carcinoma, liver cancer, colorectal cancer, and lung cancer (33–36).

However, current data about associations between CALLY index and critically ill patients were limited, whether CALLY index was an independent factor of prognosis in sepsis patients has not been determined yet. Based on the current research status, the aim of this study was to assess the role of the CALLY index in predicting the prognosis of critically ill patients with sepsis in the ICU.

## Methods

### Study population

**Figure 1:**
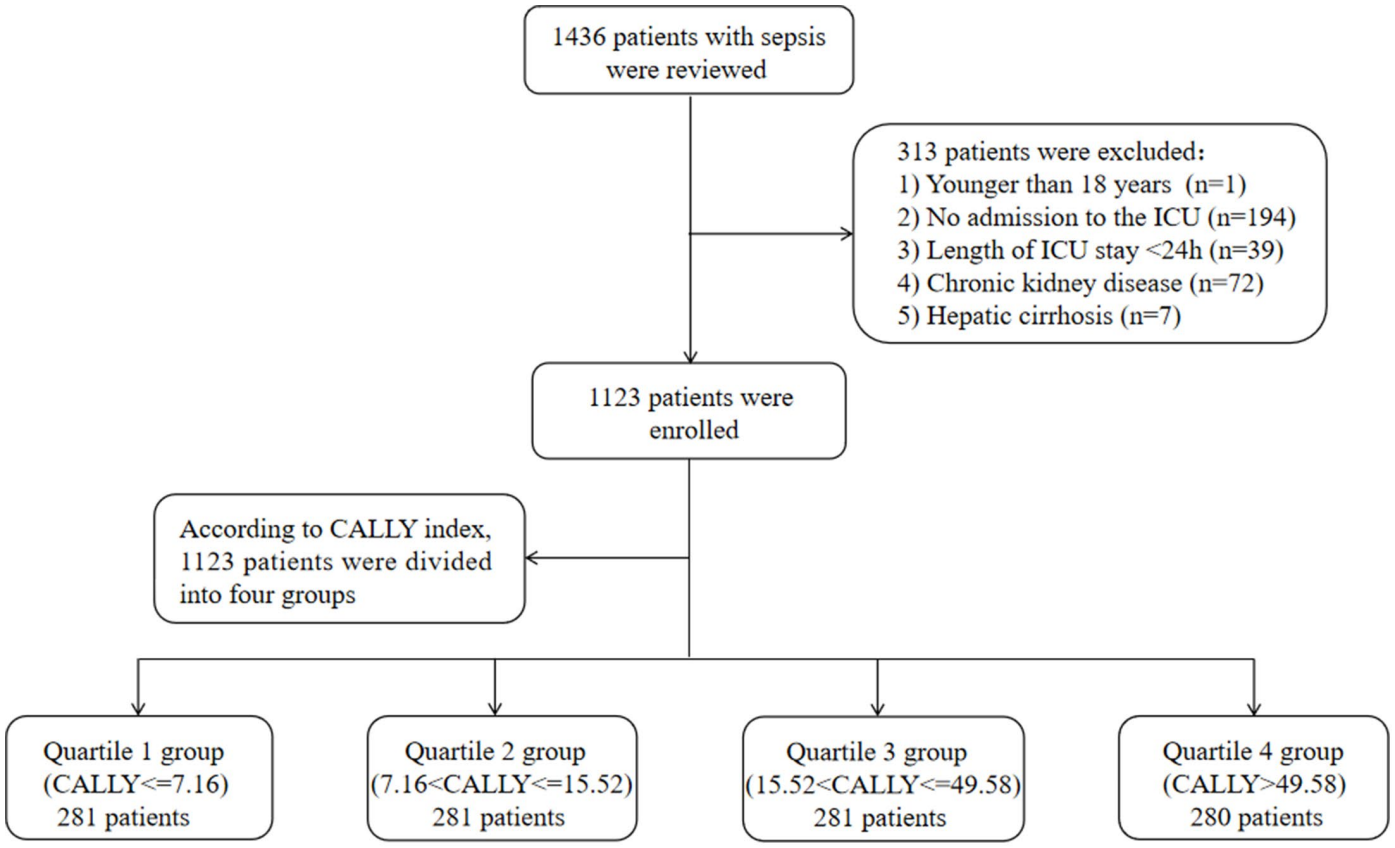
Flow of included patients through the trial. CALLY index, CRP-albumin-lymphocyte index; ICU, Intensive Care Unit.

This retrospective cohort study included a total of 1,436 patients diagnosed with sepsis between January 2015 and November 2023. The anonymized clinical data were collected from the Affiliated Hospital of Jiangsu University. The study employed the sepsis 3.0 criteria, which defined sepsis as a Sequential Organ Failure Assessment (SOFA) score ≥ 2 and documented or suspected infection, to diagnose sepsis (1). In order to ensure consistency, only the first admission for patients admitted multiple times was considered for the study. Several exclusion criteria were applied, including patients under the age of 18, patients with chronic kidney disease (CKD), patients with hepatic cirrhosis, patients not admitted to the ICU, and patients with an ICU length of stay less than 24 h. After applying these criteria, a total of 1,123 patients were enrolled in the study. These patients were then grouped into four quartiles based on the CALLY index calculated on the first day of their ICU stay. The patient screening process is illustrated in Figure 1. The study protocol was approved by the ethics committee of the Affiliated Hospital of Jiangsu University (No. KY2023K1007). To protect their privacy, written informed consent was obtained from all participants for the use of their clinical data while ensuring that their personal information remains undisclosed.

### Variable extraction

The potential variables for this study were extracted from the electronic medical record system and can be categorized into six main groups. (1) Demographics: This included variables such as age, gender, body mass index (BMI), and smoking status. (2) Comorbidities: This group included pre-existing conditions like hypertension, diabetes, coronary artery disease, chronic obstructive pulmonary disease (COPD), and cerebral infarction. (3) Infection pathogens: This category included information on the type of infection pathogen present, such as gram-positive bacterial infection, gram-negative bacterial infection, fungal infection, and viral infection. (4) infection locations: This included infection locations such as multisite infection, lower respiratory infection, gastrointestinal infection, intra-abdominal infection, genitourinary tract infection, bacteremia, and skin and soft tissue infection; (5) Laboratory indicators: This group encompassed various laboratory test results and includes variables like white blood cell (WBC), neutrophil (Neu), lymphocyte (Lym), monocyte (Mon), hemoglobin (Hb), platelet (PLT), C-reactive protein (CRP), total bilirubin (Tbil), alanine transaminase (ALT), aspartate aminotransferase (AST), albumin, glucose, creatinine, blood urea nitrogen (BUN), uric acid, D-dimer, potassium, and lactate. (6) Severity of illness scores at ICU admission: This included scores such as the Acute Physiology and Chronic Health Evaluation II (APACHE II) score and SOFA score. (7) Treatments: This group included information on specific treatments administered to the patients, such as the use of continuous renal replacement therapy (CRRT), vasoactive drugs, and invasive ventilation. All laboratory indicators and disease severity scores were extracted within the first 24 h after the patient entered the ICU. The follow-up period for each patient began on the date of admission and ended on the date of death or discharge. The formula for calculating CALLY index was previously described as [100*Albumin(g/L)*Lymphocyte count(10^9^/L)]/[CRP(mg/L)].

### Primary outcome and secondary outcomes

In this study, the primary outcome was the mortality rate at 30 and 60 days. The researchers also examined several secondary outcomes, including the occurrence of acute kidney injury (AKI), length of stay in the ICU and hospital, ICU mortality, and hospital mortality. AKI was defined using the Kidney Disease: Improving Global Outcomes (KDIGO) guidelines (37).

### Statistical analysis

Continuous variables were reported as mean with standard deviation (SD) or median with interquartile range (IQR), while categorical variables were presented as quantity and frequency (%). To compare continuous variables, Student’s *t*-test or ANOVA test was used, and for categorical variables, the Pearson chi-square test or Fisher’s exact test was employed. Kaplan–Meier survival analysis was used to estimate the incidence rate of 30-/60-day mortality among groups based on different levels of the CALLY index. The differences between these groups were assessed using log-rank tests.

Cox proportional hazards models were used to calculate the hazard ratio (HR) and 95% confidence interval (CI) between the CALLY index and endpoints, and also adjusted for some models. Confounding variables were selected based on a significance level of *p* < 0.05 in the univariate analysis. Additionally, clinically relevant and prognosis-associated variables were also included in the multivariate model: model 1: unadjusted; model 2: adjusted for age, gender, BMI, smoking, hypertension, diabetes, WBC, APACHE II score, and SOFA score; model 3: adjusted for age, gender, BMI, smoking, hypertension, diabetes, WBC, creatinine, BUN, uric acid, D-dimer, potassium, lactate, APACHE II score, SOFA score, and invasive ventilation. Meanwhile, the association between the CALLY index and the incidence of AKI was investigated through logistic regression models with different degrees of covariate adjustment. Model 1 was a univariate analysis without adjusting for any covariates. Model 2 adjusted for age, gender, BMI, smoking, hypertension, diabetes, WBC, APACHE II score, and SOFA score. Model 3 adjusted for age, gender, BMI, smoking, hypertension, diabetes, WBC, uric acid, D-dimer, potassium, lactate, APACHE II score, SOFA score, and invasive ventilation. In addition, we employed receiver operating characteristic (ROC) analysis to estimate the predictive value of the CALLY index for 30-/60-day mortality and the incidence of AKI. The CALLY index was included in the models as either continuous variables or ordinal variables, with the reference group being the first quartile of the CALLY index. The *p* values for trends were calculated based on the quartile level. Furthermore, we conducted further stratified analyses based on age (≤65 or > 65), gender (male or female), hypertension (yes or no), diabetes (yes or no), smoking (yes or no), and AKI (yes or no) to assess the consistency of the prognostic impact of the CALLY index on 30-/60-day mortality. Additionally, we investigated whether the CALLY index was associated with the occurrence of AKI in subgroups stratified by age (≤65 or > 65), gender (male or female), hypertension (yes or no), diabetes (yes or no), smoking (yes or no), and lactate level (≤2.0 or > 2.0). We also performed further testing to evaluate the interaction between the CALLY index and the stratified variables A double-sided *p* value of less than 0.05 was considered statistically significant. The statistical analyses were performed using IBM SPSS 26.0 and GraphPad Prism 10.0 software.

## Results

### Study population

In this study, a total of 1,123 critically ill patients with sepsis were enrolled. The median age of the patients was 75 years (IQR: 65–84), and 707 (63.0%) were male. Among the enrolled patients, 229 (20.4%) were smokers, and the median BMI was 22.49 kg/m^2^ (IQR: 20.08–25.21). Hypertension was the most prevalent comorbidity, affecting 51.3% of the patients, followed by diabetes (27.5%), cerebral infarction (14.3%), coronary artery disease (10.3%), and COPD (7.7%). The median CALLY index for all included participants was 15.52 (IQR: 7.16–49.58). Out of the 1,123 patients included in the study, 78 (6.9%) required CRRT, and 748 (66.6%) received vasoactive drugs, with 752 (67.0%) requiring invasive ventilation. During the hospital admission, the 30- and 60-day mortality rates were 28.1 and 33.4%, respectively, while the incidence of AKI was 45.6% (Table 1).

**Table 1:** Characteristics and outcomes of participants categorized by CALLY index.

Variables	Overall	Q1 group (CALLY ≤ 7.16)	Q2 group (7.16 < CALLY ≤ 15.52)	Q3 group (15.52 < CALLY ≤ 49.58)	Q4 group (CALLY > 49.58)	*p* value
N	1,123	281	281	281	280	
Age, years	75 (65–84)	75 (65–83)	76 (66–84)	76 (66–85)	75 (64–85)	0.633
Male, *n* (%)	707 (63.0)	169 (60.1)	188 (66.9)	180 (64.1)	170 (60.7)	0.310
BMI, kg/m^2^	22.49 (20.08–25.21)	22.40 (20.04–24.53)	22.49 (20.09–25.37)	22.76 (20.24–25.71)	22.49 (19.99–25.36)	0.392
Smoking, *n* (%)	229 (20.4)	62 (22.1)	71 (25.3)	46 (16.4)	50 (17.9)	0.038
Comorbidities, *n* (%)						
Hypertension	579 (51.3)	134 (47.7)	154 (54.8)	154 (54.8)	137 (48.9)	0.184
Diabetes	309 (27.5)	73 (26.0)	88 (31.3)	74 (26.3)	74 (26.4)	0.435
Coronary artery disease	116 (10.3)	27 (9.6)	38 (13.5)	27 (9.6)	24 (8.6)	0.230
COPD	87 (7.7)	12 (4.3)	19 (6.8)	25 (8.9)	31 (11.1)	0.019
Cerebral infarction	161 (14.3)	36 (12.8)	37 (13.2)	47 (16.7)	41 (14.6)	0.537
Infection pathogens, *n* (%)						
Gram-positive bacteria	136 (12.1)	38 (13.5)	32 (11.4)	31 (11.0)	35 (12.5)	0.798
Gram-negative bacteria	335 (29.8)	98 (34.9)	95 (33.8)	82 (29.2)	60 (21.4)	0.002
Fungus	77 (6.9)	23 (8.2)	25 (8.9)	20 (7.1)	9 (3.2)	0.038
Virus	60 (5.3)	10 (3.6)	21 (7.5)	18 (6.4)	11 (3.9)	0.110
Infection sites, *n* (%)						
Multisite infection	120 (10.7)	38 (13.5)	38 (13.5)	34 (12.1)	10 (3.6)	<0.001
Lower respiratory infection	436 (38.8)	93 (33.1)	119 (42.3)	110 (39.1)	114 (40.7)	0.123
Gastrointestinal infection	11 (1.0)	5 (1.8)	3 (1.1)	1 (0.4)	2 (0.7)	0.361
Intra-abdominal infection	392 (34.9)	100 (35.6)	93 (33.1)	92 (32.7)	107 (38.2)	0.495
Genitourinary tract infection	69 (6.1)	21 (7.5)	12 (4.3)	20 (7.1)	16 (5.7)	0.373
Bacteremia	10 (0.9)	4 (1.4)	1 (0.4)	2 (0.7)	3 (1.1)	0.568
Skin and soft tissue infection	85 (7.6)	21 (7.5)	15 (5.3)	21 (7.5)	28 (10.0)	0.224
Laboratory tests						
WBC *10^9^/L	11.4 (7.4–17.1)	10.3 (6.6–16.0)	13.3 (8.2–18.3)	11.2 (7.9–16.6)	11.2 (7.4–16.6)	0.003
Neu *10^9^/L	10.1 (6.3–15.5)	9.5 (6.1–15.6)	12.5 (6.9–16.9)	9.8 (6.7–15.2)	9.3 (6.1–14.6)	0.003
Lym *10^9^/L	0.6 (0.3–0.9)	0.3 (0.2–0.4)	0.5 (0.3–0.7)	0.8 (0.5–1.0)	0.9 (0.6–1.4)	<0.001
Mon *10^9^/L	0.4 (0.2–0.7)	0.3 (0.2–0.5)	0.4 (0.2–0.6)	0.5 (0.3–0.7)	0.5 (0.2–0.8)	<0.001
Hb, g/dL	115 (97–130)	107 (91–124)	112 (96–128)	117 (100–132)	119 (103–137)	<0.001
PLT *10^9^/L	149 (95–214)	115 (77–166)	138 (87–207)	160 (103–227)	189 (139–256)	<0.001
CRP, mg/L	104.2 (42.0–163.2)	183.7 (137.1–236.3)	137.4 (95.2–171.1)	86.7 (56.7–118.3)	12.3 (4.0–32.8)	<0.001
CALLY index	15.52 (7.16–49.58)	4.13 (2.86–5.64)	10.49 (8.52–12.84)	25.63 (19.71–33.45)	199.07 (89.45–653.83)	<0.001
Tbil, μmol/L	17.4 (10.9–28.2)	18.8 (12.2–32.2)	18.4 (11.8–30.9)	17.8 (11.6–26.4)	13.9 (8.4–23.3)	<0.001
ALT, U/L	32.0 (21.0–56.0)	33.0 (22.0–59.5)	32.0 (21.0–61.0)	30.0 (20.4–53.5)	32.0 (21.0–50.0)	0.475
AST, U/L	38.1 (23.9–73.0)	47.0 (26.0–84.0)	38.0 (24.5–84.9)	35.3 (23.0–67.3)	33.5 (22.0–63.6)	0.001
Albumin, g/L	28.2 (24.2–33.2)	25.5 (21.8–29.4)	27.0 (23.2–32.1)	29.0 (25.6–33.7)	32.2 (28.3–36.9)	<0.001
Glucose, mmol/L	8.2 (6.6–11.8)	8.59 (6.68–12.78)	8.52 (6.52–12.70)	8.11 (3.57–11.61)	7.88 (6.52–10.14)	0.084
Creatinine, μmol/L	92.6 (63.7–153.1)	121.3 (75.1–199.1)	109.6 (65.8–167.9)	85.5 (61.8–130.2)	76.6 (56.6–123.7)	<0.001
BUN, mmol/L	8.89 (6.04–13.95)	11.64 (7.12–18.70)	10.39 (6.73–14.90)	7.84 (5.55–11.09)	7.17 (5.24–10.61)	<0.001
Uric acid, μmol/L	286.9 (192.3–411.7)	306.4 (204.8–462.4)	282.7 (184.2–414.6)	283.9 (196.2–376.5)	276.1 (190.2–400.6)	0.055
D-dimer, mg/L	4.2 (2.1–8.4)	5.0 (2.7–9.4)	5.3 (2.5–9.3)	3.7 (1.9–7.5)	3.4 (1.6–7.2)	<0.001
Potassium, mmol/L	3.7 (3.3–4.2)	3.7 (3.3–4.2)	3.7 (3.3–4.2)	3.6 (3.2–4.0)	3.8 (3.3–4.2)	0.034
Lactate, mmol/L	2.1 (1.4–3.6)	2.2 (1.6–3.7)	2.1 (1.5–4.0)	2.1 (1.3–3.3)	2.0 (1.3–3.4)	0.009
Severity scoring						
APACHE II score	25 (19–30)	27 (20–31)	25 (20–31)	24 (19–29)	25 (18–30)	0.029
SOFA score	12 (10–14)	13 (10–15)	12 (10–14)	12 (9–14)	12 (9–14)	<0.001
Treatments						
CRRT, *n* (%)	78 (6.9)	27 (9.6)	24 (8.5)	14 (5.0)	13 (4.6)	0.043
Vasoactive drug, *n* (%)	748 (66.6)	229 (81.5)	197 (70.1)	175 (62.3)	147 (52.4)	<0.001
Invasive ventilation, *n* (%)	752 (67.0)	195 (69.4)	178 (63.3)	184 (65.5)	195 (69.6)	0.308
Endpoints						
30-day mortality, *n* (%)	316 (28.1)	103 (36.7)	93 (33.1)	74 (26.3)	46 (16.4)	<0.001
60-day mortality, *n* (%)	375 (33.4)	118 (42.0)	111 (39.5)	88 (31.3)	58 (20.7)	<0.001
AKI, *n* (%)	512 (45.6)	171 (60.9)	140 (49.8)	110 (39.1)	91 (32.5)	<0.001
Length of ICU stay, days	6 (3–12)	6 (3–12)	7 (4–13)	6 (3–11)	5 (2–11)	<0.001
Length of hospital stay, days	16 (11–25)	17 (9–27)	17 (11–27)	16 (10–24)	17 (11–25)	0.240
ICU mortality, *n* (%)	358 (31.9)	113 (40.2)	108 (38.4)	81 (28.8)	56 (20.2)	<0.001
Hospital mortality, *n* (%)	379 (33.7)	120 (42.7)	113 (40.2)	87 (31.0)	59 (21.1)	<0.001

CALLY index, CRP-albumin-lymphocyte index; BMI, Body mass index; COPD, Chronic obstructive pulmonary disease; WBC, White blood cell count; Neu, Neutrophil; Lym, Lymphocyte; Mon, Monocyte; Hb, Hemoglobin; PLT, Platelet; CRP, C-reactive protein; Tbil, Total bilirubin; ALT, Alanine transaminase; AST, Aspartate aminotransferase; BUN, Blood urea nitrogen; APACHE II, Acute physiology and chronic health evaluation II; SOFA, Sequential organ failure assessment; CRRT, Continuous renal replacement therapy; AKI, Acute kidney injury; ICU, Intensive Care Unit.

### Baseline characteristics

The baseline characteristics of critically ill patients with sepsis, divided according to the CALLY index quartiles, were presented in Table 1. The enrolled individuals were categorized into four groups based on their ICU admission CALLY index levels: Q1 (CALLY index ≤ 7.16), Q2 (7.16 < CALLY index ≤ 15.52), Q3 (15.52 < CALLY index ≤ 49.58), and Q4 (CALLY index > 49.58). The median values of the CALLY index for each quartile were 4.13 (IQR: 2.86–5.64), 10.49 (IQR: 8.52–12.84), 25.63 (IQR: 19.71–33.45), and 199.07 (IQR: 89.45–653.83), respectively.

Patients in the highest quartile of the CALLY index generally had a lower BMI, a lower prevalence of smoking, a lower level of WBC, Neu, CRP, AST, glucose, creatinine, BUN, D-dimer, and lactate, a lower severity of illness score on ICU admission, and a lower proportion of individuals requiring CRRT and vasoactive drugs, compared to the lower quartiles (*p* < 0.05). Conversely, Lym, Mon, Hb, PLT, and albumin levels were significantly higher in the highest quartile (*p* < 0.001). Comparing individuals in the higher quartile of the CALLY index to those in the lower quartiles, it was found that the former had lower 30-day mortality (16.4 vs. 26.3% vs. 33.1 vs. 36.7%, *p* < 0.001), 60-day mortality (20.7 vs. 31.3% vs. 39.5 vs. 42.0%, *p* < 0.001), AKI occurrence (32.5 vs. 39.1% vs. 49.8 vs. 60.9%, *p* < 0.001), ICU mortality (20.2 vs. 28.8% vs. 38.4 vs. 40.2%, *p* < 0.001), hospital mortality (21.1 vs. 31.0% vs. 40.2 vs. 42.7%, *p* < 0.001), and shorter ICU length of stay (5 vs. 6 vs. 7 vs. 6 days, *p* < 0.001). Further analysis comparing Q1-3 group to Q4 demonstrated similar results, indicating a strong association between the CALLY index quartiles and poor outcomes (Supplementary Table S1).

**Figure 2:**
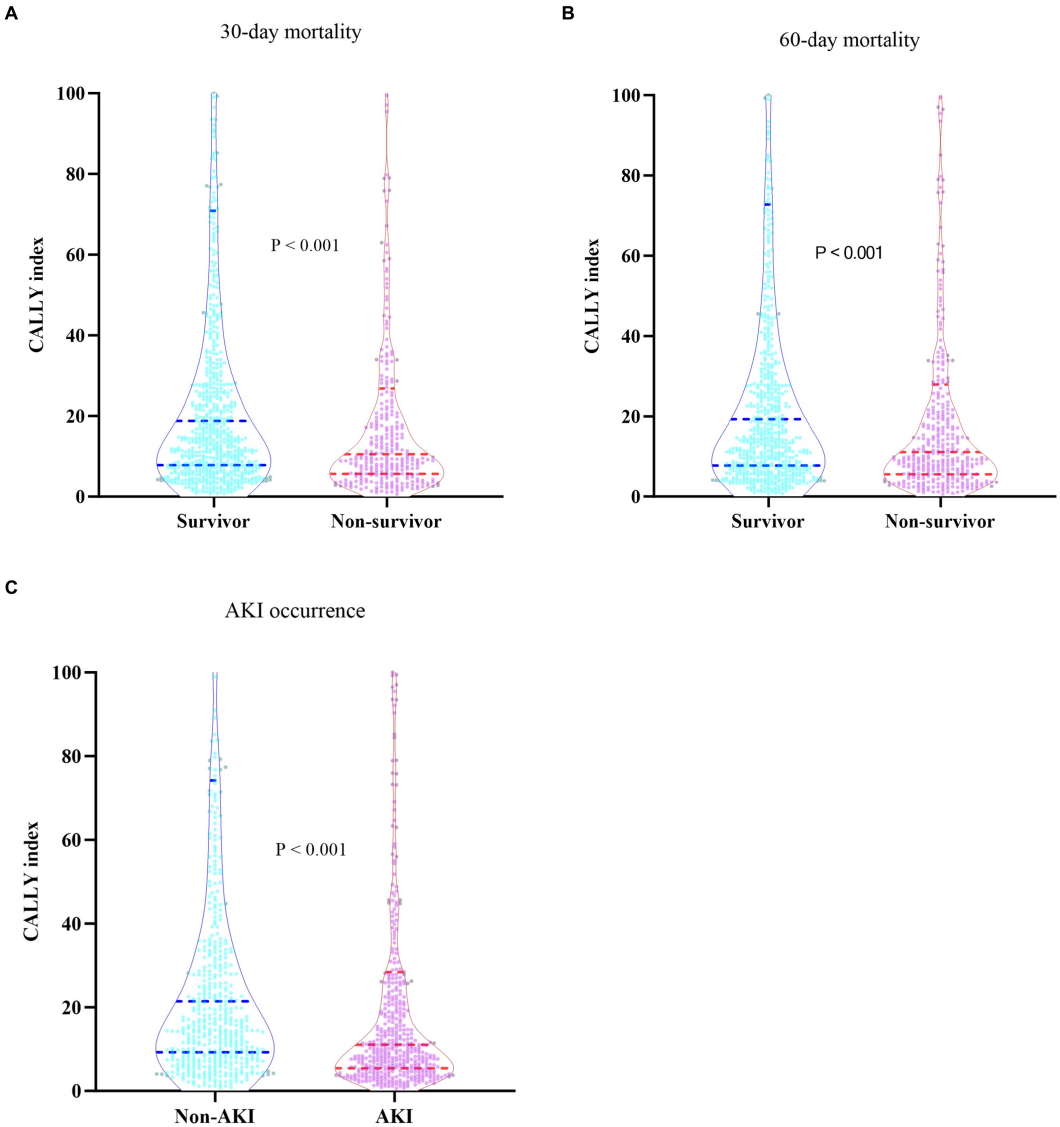
**(A)** Boxplots of the CALLY index showing the distribution in the Survivor group and Non-survivor group at 30 days. **(B)** Boxplots of the CALLY index showing the distribution in the Survivor group and Non-survivor group at 60 days. **(C)** Boxplots of the CALLY index showing the distribution in the Non-AKI group and AKI group. CALLY index, CRP-albumin-lymphocyte index; AKI, Acute kidney injury.

Table 2 presented the differences in baseline characteristics between Survivors and Non-survivors during the hospital stay. Patients in the Non-survivor group were more likely to be older and had a higher prevalence of coronary artery disease, COPD, cerebral infarction, gram-negative bacteria, fungus, virus infections, and lower respiratory infection. They also had higher levels of WBC, Neu, CRP, ALT, AST, glucose, creatinine, BUN, uric acid, D-dimer, potassium, and lactate, and lower levels of BMI, Lym, Mon, Hb, PLT, CALLY index, and albumin. Additionally, the Non-survivor group had a higher proportion of individuals requiring CRRT, vasoactive drugs, and invasive ventilation, as well as higher severity of illness scores. The CALLY index levels in the Non-survivor group were significantly lower than those in the Survivor group (11.14 vs. 19.41, *p* < 0.001). Figures 2A,B depicted the distribution of the CALLY index stratified by the mortality status of all-cause death at 30 and 60 days, respectively.

**Table 2:** Baseline characteristics of the Survivor and Non-survivor groups.

Variables	Overall	Survivor group	Non-survivor group	*p* value
*N*	1,123	744	379	
Age, years	75 (65–84)	73 (62–83)	79 (71–86)	<0.001
Male, *n* (%)	707 (63.0)	456 (61.3)	251 (66.2)	0.105
BMI, kg/m^2^	22.49 (20.08–25.21)	22.86 (20.48–25.63)	22.03 (19.53–24.24)	<0.001
Smoking, *n* (%)	229 (20.4)	142 (19.1)	87 (23.0)	0.123
Comorbidities, *n* (%)				
Hypertension	579 (51.3)	368 (49.5)	211 (55.7)	0.049
Diabetes	309 (27.5)	198 (26.6)	111 (29.3)	0.343
Coronary artery disease	116 (10.3)	59 (7.9)	57 (15.0)	<0.001
COPD	87 (7.7)	42 (5.6)	45 (11.9)	<0.001
Cerebral infarction	161 (14.3)	90 (12.1)	71 (18.7)	0.003
Infection pathogens, *n* (%)				
Gram-positive bacteria	136 (12.1)	87 (11.7)	49 (12.9)	0.549
Gram-negative bacteria	335 (29.8)	207 (27.8)	128 (33.8)	0.039
Fungus	77 (6.9)	32 (4.3)	45 (11.9)	<0.001
Virus	60 (5.3)	22 (3.0)	38 (10.0)	<0.001
Infection sites, *n* (%)				
Multisite infection	120 (10.7)	76 (10.2)	44 (11.6)	0.474
Lower respiratory infection	436 (38.8)	210 (28.2)	226 (59.6)	<0.001
Gastrointestinal infection	11 (1.0)	8 (1.1)	3 (0.8)	0.648
Intra-abdominal infection	392 (34.9)	319 (42.9)	73 (19.3)	<0.001
Genitourinary tract infection	69 (6.1)	58 (7.8)	11 (2.9)	0.001
Bacteremia	10 (0.9)	8 (1.1)	2 (0.5)	0.356
Skin and soft tissue infection	85 (7.6)	65 (8.7)	20 (5.3)	0.038
Laboratory tests				
WBC *10^9^/L	11.4 (7.4–17.1)	11.0 (7.2–16.7)	12.5 (7.9–17.8)	0.008
Neu *10^9^/L	10.1 (6.3–15.5)	9.5 (6.1–15.0)	11.5 (7.0–16.7)	0.001
Lym *10^9^/L	0.6 (0.3–0.9)	0.6 (0.4–1.0)	0.4 (0.3–0.7)	<0.001
Mon *10^9^/L	0.4 (0.2–0.7)	0.4 (0.2–0.7)	0.4 (0.2–0.7)	0.546
Hb, g/dL	115 (97–130)	119 (101–133)	106 (88–124)	<0.001
PLT *10^9^/L	149 (95–214)	163 (105–227)	130 (77–197)	<0.001
CRP, mg/L	104.2 (42.0–163.2)	102.6 (32.2–161.1)	107.6 (54.0–171.9)	0.014
CALLY index	15.52 (7.16–49.58)	19.41 (7.85–73.07)	11.14 (5.64–28.00)	<0.001
Tbil, μmol/L	17.4 (10.9–28.2)	16.9 (10.9–27.4)	18.1 (10.8–29.9)	0.229
ALT, U/L	32.0 (21.0–56.0)	30.9 (21.0–50.0)	35.0 (22.0–70.0)	0.002
AST, U/L	38.1 (23.9–73.0)	35.0 (22.0–63.0)	50.0 (27.5–113.0)	<0.001
Albumin, g/L	28.2 (24.2–33.2)	29.1 (24.7–33.7)	27.0 (23.3–32.2)	<0.001
Glucose, mmol/L	8.2 (6.6–11.8)	8.1 (6.6–11.3)	8.8 (6.6–12.3)	0.045
Creatinine, μmol/L	92.6 (63.7–153.1)	85.0 (60.2–132.9)	124.7 (69.8–186.2)	<0.001
BUN, mmol/L	8.89 (6.04–13.95)	8.04 (5.53–11.65)	11.31 (7.33–18.85)	<0.001
Uric acid, μmol/L	286.9 (192.3–411.7)	277.3 (189.1–375.8)	322.4 (203.6–487.9)	<0.001
D-dimer, mg/L	4.2 (2.1–8.4)	3.7 (2.0–7.2)	6.0 (2.7–10.1)	<0.001
Potassium, mmol/L	3.7 (3.3–4.2)	3.7 (3.3–4.1)	3.8 (3.3–4.3)	0.021
Lactate, mmol/L	2.1 (1.4–3.6)	1.9 (1.3–2.9)	2.8 (2.0–5.3)	<0.001
Severity scoring				
APACHE II score	25 (19–30)	24 (18–29)	28 (23–34)	<0.001
SOFA score	12 (10–14)	11 (9–14)	13 (11–15)	<0.001
Treatments				
CRRT, *n* (%)	78 (6.9)	19 (2.6)	59 (15.6)	<0.001
Vasoactive drug, *n* (%)	748 (66.6)	403 (54.2)	345 (91.0)	<0.001
Invasive ventilation, *n* (%)	752 (67.0)	413 (55.5)	339 (89.4)	<0.001
Endpoints				
AKI, *n* (%)	512 (45.6)	289 (38.8)	223 (58.8)	<0.001
Length of ICU stay, days	6 (3–12)	4 (3–9)	10 (5–18)	<0.001
Length of hospital stay, days	16 (11–25)	17 (12–26)	14 (7–25)	<0.001

CALLY index, CRP-albumin-lymphocyte index; BMI, Body mass index; COPD, Chronic obstructive pulmonary disease; WBC, White blood cell count; Neu, Neutrophil; Lym, Lymphocyte; Mon, Monocyte; Hb, Hemoglobin; PLT, Platelet; CRP, C-reactive protein; Tbil, Total bilirubin; ALT, Alanine transaminase; AST, Aspartate aminotransferase; BUN, Blood urea nitrogen; APACHE II, Acute physiology and chronic health evaluation II; SOFA, Sequential organ failure assessment; CRRT, Continuous renal replacement therapy; AKI, Acute kidney injury; ICU, Intensive Care Unit.

### Incidence rate of 30-/60-day mortality among different groups

**Figure 3:**
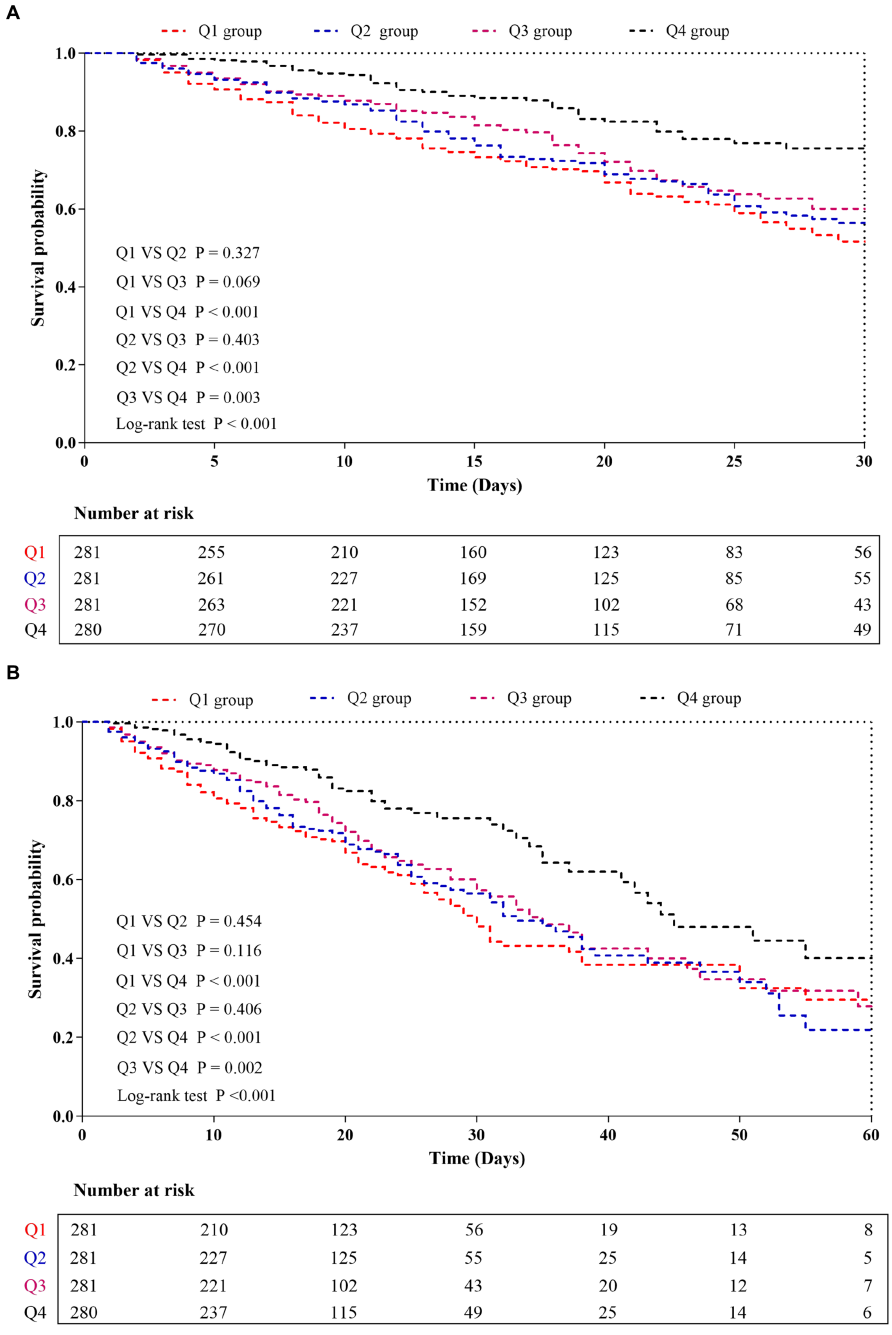
Kaplan–Meier curves showing cumulative probability of all-cause mortality according to groups at 30 days **(A)**, and 60 days **(B)**. CALLY index quartiles: Q1 group (CALLY ≤ 7.16); Q2 group (7.16 < CALLY ≤ 15.52); Q3 group (15.52 < CALLY ≤ 49.58); and Q4 group (CALLY > 49.58).

**Figure 4:**
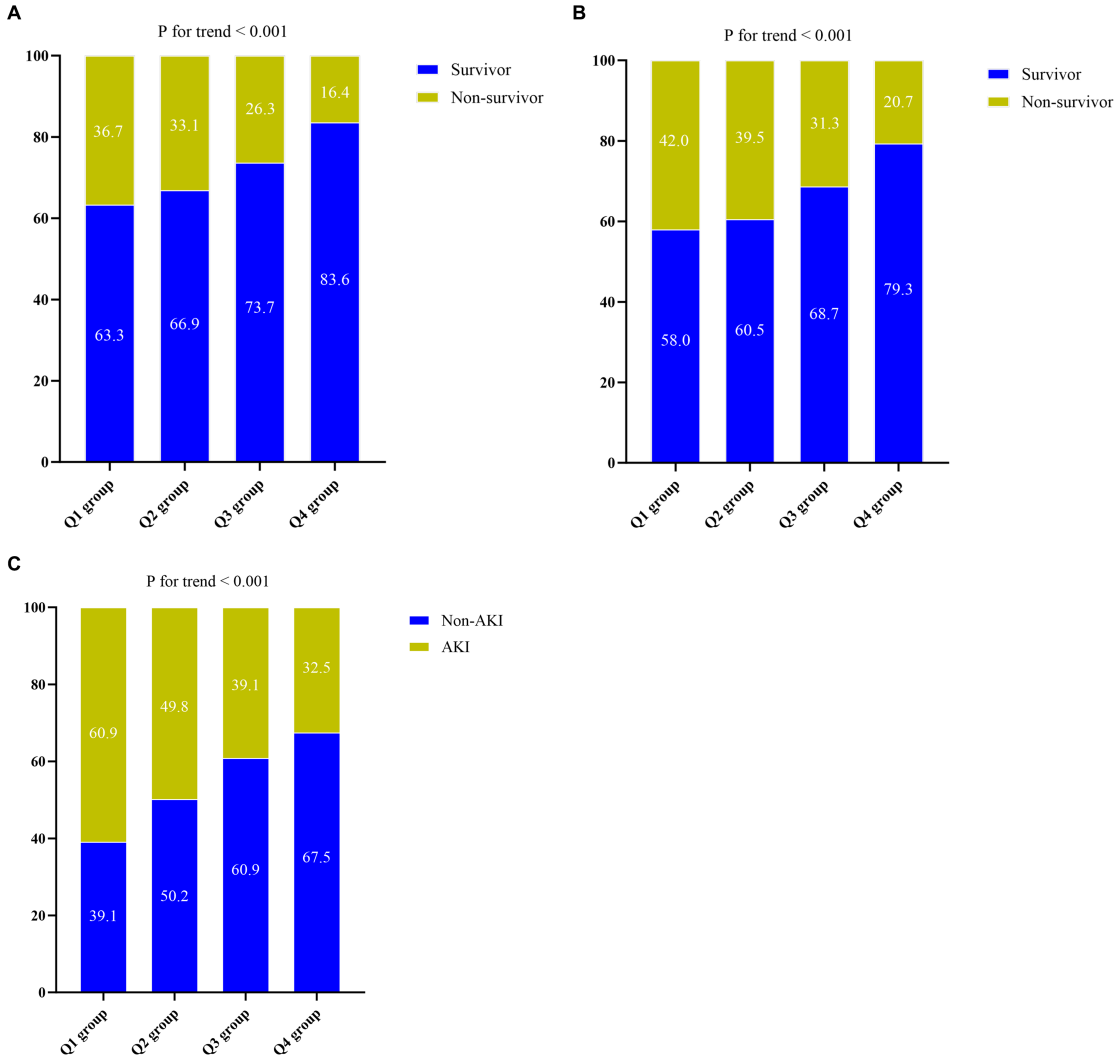
**(A)** The prevalence of 30-day mortality ratio among different quartiles of CALLY index. **(B)** The prevalence of 60-day mortality ratio among different quartiles of CALLY index. **(C)** The prevalence of AKI occurrence ratio among different quartiles of CALLY index. CALLY index quartiles: Q1 group (CALLY ≤ 7.16); Q2 group (7.16 < CALLY ≤ 15.52); Q3 group (15.52 < CALLY ≤ 49.58); Q4 group (CALLY > 49.58). CALLY index, CRP-albumin-lymphocyte index; AKI, Acute kidney injury.

**Figure 5:**
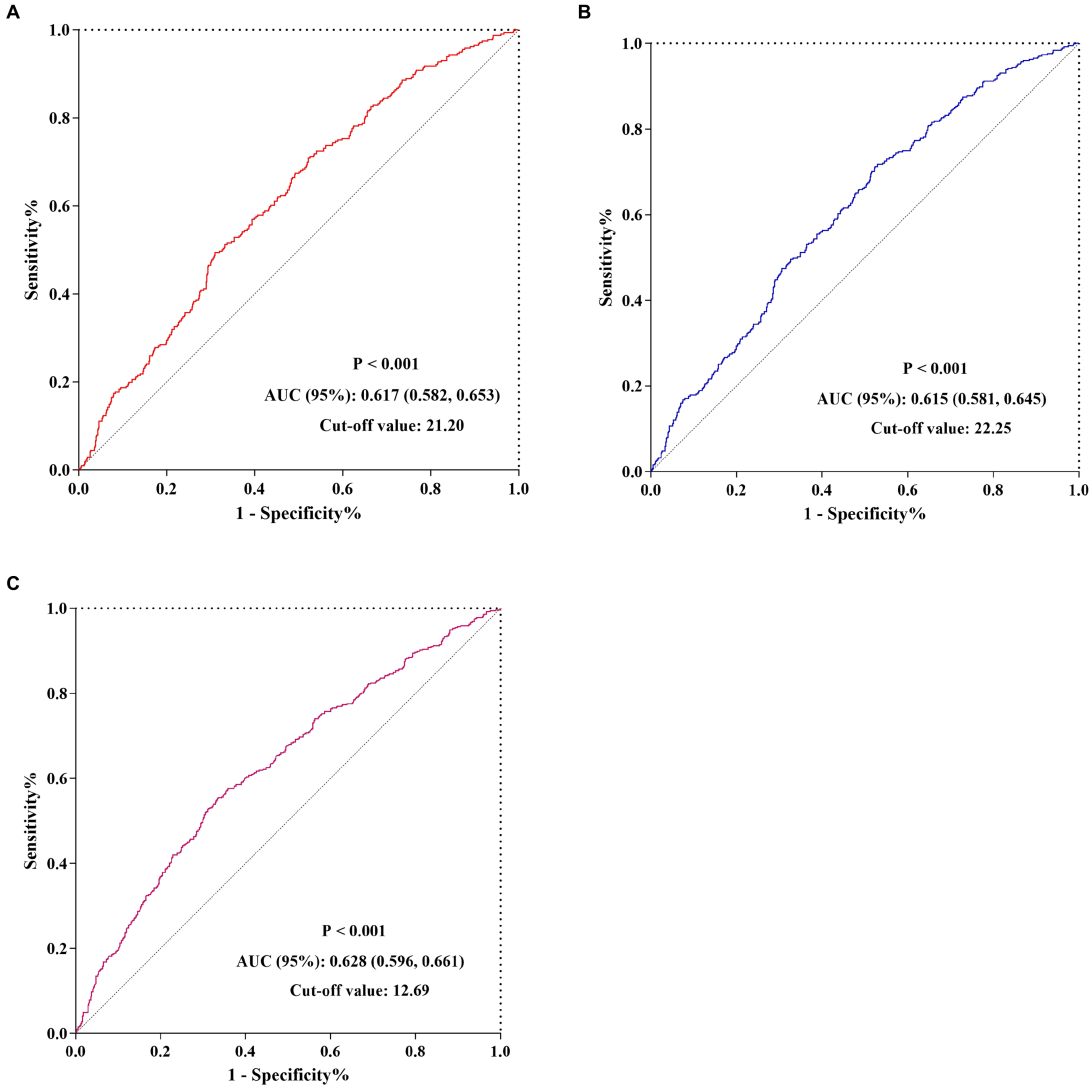
The predictive value of CALLY index for 30-/60-day mortality and AKI occurrence by ROC analysis. **(A)** The predictive value of CALLY index for 30-day mortality by ROC analysis. **(B)** The predictive value of CALLY index for 60-day mortality by ROC analysis. **(C)** The predictive value of CALLY index for AKI occurrence by ROC analysis. CALLY index, CRP-albumin-lymphocyte index; ROC, Receiver operating characteristic curve; AUC, Area under curve; AKI, Acute kidney injury.

The Kaplan–Meier survival analysis curves were used to evaluate the incidence of 30-/60-day mortality among various groups based on quartile groupings of the CALLY index. Figure 3 displayed the results, indicating that patients with a higher CALLY index had a lower risk of 30-/60-day mortality. There was a significant difference in the 30-day mortality rate among the groups (Q1: 36.7% vs. Q2: 33.1% vs. Q3: 26.3% vs. Q4: 16.4%, log-rank *p* < 0.001; Figure 3A) and in the 60-day mortality rate among the groups (Q1: 42.0% vs. Q2: 39.5% vs. Q3: 31.3% vs. Q4: 20.7%, log-rank *p* < 0.001; Figure 3B). These findings suggested a decreasing trend in mortality rates with higher CALLY index (Figures 4A,B). Furthermore, our analysis demonstrated that the CALLY index had a higher predictive power for both 30-day mortality (AUC: 0.617: 95% CI: 0.582–0.653; *p* < 0.001) and 60-day mortality (AUC: 0.615; 95% CI: 0.581–0.645; *p* < 0.001) compared to the other indicators, such as WBC, Neu, lymphocyte, CRP, albumin, APACHE II score, and SOFA score (Supplementary Table S2). The identified cut-off values for the CALLY index were 20.20 and 22.25 for 30- and 60-day mortality, respectively (Figures 5A,B).

**Figure 6:**
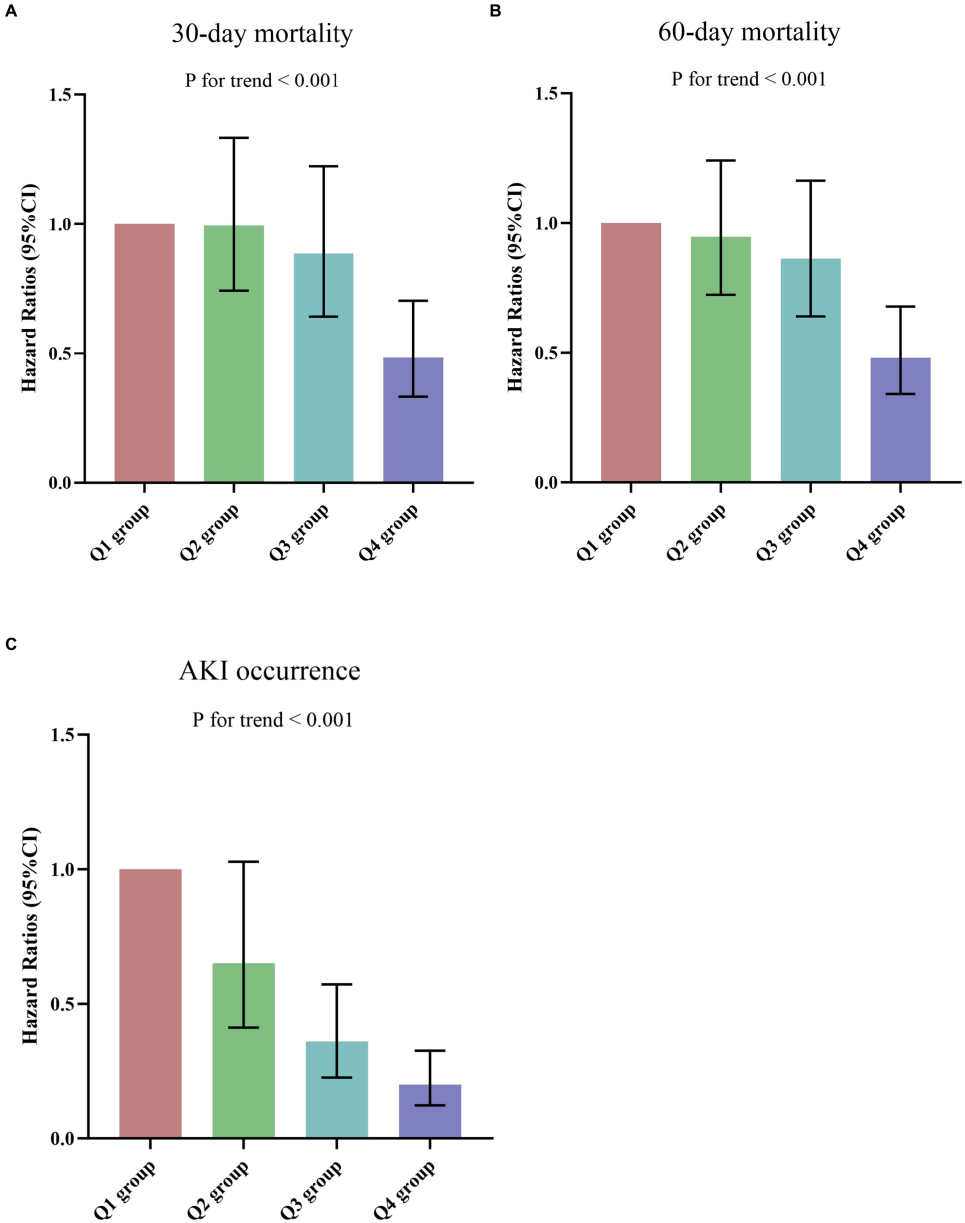
**(A,B)** Hazard ratios (95% CIs) for 30-/60-day mortality according to CALLY index quartiles after adjusting for age, gender, BMI, smoking, hypertension, diabetes, WBC, creatinine, BUN, uric acid, D-dimer, potassium, lactate, APACHE II score, SOFA score, and invasive ventilation. Error bars indicate 95% CIs. The first quartile is the reference. **(C)** Odds Ratios (95% CIs) for AKI occurrence according to CALLY index quartiles after adjusting for age, gender, BMI, smoking, hypertension, diabetes, WBC, uric acid, D-dimer, potassium, lactate, APACHE II score, SOFA score, and invasive ventilation. Error bars indicate 95% CIs. The first quartile is the reference. CALLY index quartiles: Q1 group (CALLY ≤ 7.16); Q2 group (7.16 < CALLY ≤ 15.52); Q3 group (15.52 < CALLY ≤ 49.58); Q4 group (CALLY > 49.58). CALLY index, CRP-albumin-lymphocyte index; BMI, Body mass index; WBC, White blood cell count; BUN, Blood urea nitrogen; APACHE II, Acute physiology and chronic health evaluation II; SOFA, Sequential organ failure assessment; AKI, Acute kidney injury.

The results of the univariate COX regression analysis for the risk of all-cause death in critically ill patients with sepsis were presented in Supplementary Table S3. Variables that showed significance in univariate analysis (*p* < 0.05), as well as factors suggested by clinicians and based on clinical experience, were included as independent variables in the COX regression analysis. The influential factors identified in the analysis were age, BMI, WBC, CALLY index, creatinine, BUN, uric acid, D-dimer, lactate, APACHE II score, SOFA score, and invasive ventilation. To assess the association between the CALLY index and 30-day mortality, multivariate Cox proportional hazards analysis was conducted. The results indicated that the CALLY index was a significant risk factor in the unadjusted model [HR (95% CI): 0.961 (0.930–0.994); *p* = 0.022], partly adjusted model [HR (95% CI): 0.967 (0.937–0.998); *p* = 0.035], and fully adjusted model [HR (95% CI): 0.965 (0.935–0.997); *p* = 0.030] when considering the CALLY index as a continuous variable. When the CALLY index was treated as a nominal variable, patients in the higher quartiles had a significantly lower risk of 30-day mortality compared to those in the lowest quartile in each of the Cox proportional hazards models: unadjusted model [HR (95% CI): 0.441 (0.311–0.624); *p* < 0.001], partly adjusted model [HR (95% CI): 0.470 (0.330–0.669); *p* < 0.001], and fully adjusted model [HR (95% CI): 0.483 (0.332–0.703); *p* < 0.001]. This suggested a decreasing trend in mortality risk with increasing CALLY index values (Table 3; Figure 6A). Similar results were observed in the multivariate Cox proportional hazards analysis of the CALLY index and 60-day mortality (Table 3; Figure 6B).

**Table 3:** Cox proportional hazards models for 30-/60-day mortality.

Variables	Model 1			Model 2			Model 3		
	HR (95% CI)	*p* value	*p* for trend	HR (95% CI)	*p* value	*p* for trend	HR (95% CI)	*p* value	*p* for trend
30-day mortality									
Continuous variable per unit	0.961 (0.930–0.994)	0.022		0.967 (0.937–0.998)	0.035		0.965 (0.935–0.997)	0.030	
Quartile^a^			<0.001			<0.001			<0.001
Q1 group	Ref			Ref			Ref		
Q2 group	0.867 (0.655–1.148)	0.320		0.872 (0.655–1.160)	0.346		0.994 (0.742–1.333)	0.970	
Q3 group	0.760 (0.564–1.025)	0.072		0.812 (0.596–1.105)	0.185		0.886 (0.642–1.223)	0.462	
Q4 group	0.441 (0.311–0.624)	<0.001		0.470 (0.330–0.669)	<0.001		0.483 (0.332–0.703)	<0.001	
60-day mortality									
Continuous variable per unit	0.967 (0.939–0.996)	0.025		0.969 (0.942–0.998)	0.033		0.969 (0.941–0.997)	0.032	
Quartile^a^			<0.001			<0.001			<0.001
Q1 group	Ref			Ref			Ref		
Q2 group	0.903 (0.697–1.170)	0.441		0.870 (0.668–1.133)	0.301		0.947 (0.723–1.241)	0.695	
Q3 group	0.802 (0.608–1.057)	0.117		0.806 (0.605–1.073)	0.139		0.863 (0.640–1.163)	0.333	
Q4 group	0.486 (0.355–0.666)	<0.001		0.465 (0.335–0.644)	<0.001		0.481 (0.341–0.678)	<0.001	

Model 1: unadjusted. Model 2: adjusted for age, gender, BMI, smoking, hypertension, diabetes, WBC, APACHE II score, and SOFA score. Model 3: adjusted for age, gender, BMI, smoking, hypertension, diabetes, WBC, creatinine, BUN, uric acid, D-dimer, potassium, lactate level, APACHE II score, SOFA score, and invasive ventilation. ^a^CALLY index: Q1 group (CALLY ≤ 7.16); Q2 group (7.16 < CALLY ≤ 15.52); Q3 group (15.52 < CALLY ≤ 49.58); Q4 group (CALLY > 49.58). CALLY index, CRP-albumin-lymphocyte index; BMI, Body mass index; WBC, White blood cell count; BUN, Blood urea nitrogen; APACHE II, Acute physiology and chronic health evaluation II; SOFA, Sequential organ failure assessment; AKI, Acute kidney injury.

We conducted stratified analyses to examine the relationship between the CALLY index and 30-day mortality, considering potential modifiers such as age (≤65 or > 65), gender (male or female), hypertension (yes or no), diabetes (yes or no), smoking (yes or no), and AKI (yes or no) (Table 4). The results showed that the CALLY index was significantly associated with a decreased risk of 30-day mortality in the subgroup of those >65 years [HR (95%CI): 0.965 (0.933–0.997); *p* = 0.035] and those without smoking [HR (95%CI): 0.955 (0.915–0.996); *p* = 0.033]. Similarly, in the stratified analyses of 60-day mortality, the CALLY index demonstrated a significant association with a lower risk of 60-day mortality in the subgroups of those aged >65 years [HR (95%CI): 0.967 (0.938–0.997); *p* = 0.034], male [HR (95%CI): 0.930 (0.867–0.998); *p* = 0.043], those with diabetes [HR (95%CI): 0.755 (0.577–0.986); *p* = 0.039], and those without smoking [HR (95%CI): 0.965 (0.932–0.998); *p* = 0.041] (Table 5).

**Table 4:** Subgroup analysis regarding the influence of different CALLY index in the 30-day mortality.

Subgroups	No. 30-day mortality/No. patients	HR (95% CI)	*p* value	*p* for interaction
Age				0.439
>65	266/840	0.965 (0.933–0.997)	0.035	
≤65	50/283	0.892 (0.734–1.083)	0.247	
Gender				0.346
Male	202/707	0.940 (0.881–1.003)	0.062	
Female	114/416	0.974 (0.937–1.013)	0.190	
Hypertension				0.230
Yes	173/579	0.973 (0.942–1.006)	0.105	
No	143/544	0.922 (0.849–1.001)	0.053	
Diabetes				0.094
Yes	90/309	0.755 (0.562–1.014)	0.062	
No	226/814	0.976 (0.947–1.006)	0.111	
Smoking				0.516
Yes	78/230	0.978 (0.928–1.032)	0.419	
No	238/893	0.955 (0.915–0.996)	0.033	
AKI				0.688
Yes	195/512	0.974 (0.932–1.018)	0.249	
No	121/611	0.962 (0.918–1.009)	0.113	

CALLY index, CRP-albumin-lymphocyte index; AKI, Acute kidney injury.

**Table 5:** Subgroup analysis regarding the influence of different CALLY index in the 60-day mortality.

Subgroups	No. 60-day mortality/No. patients	HR (95% CI)	*p* value	*p* for interaction
Age				0.881
>65	314/840	0.967 (0.938–0.997)	0.034	
≤65	61/283	0.958 (0.868–1.058)	0.399	
Gender				0.139
Male	246/707	0.930 (0.867–0.998)	0.043	
Female	129/416	0.984 (0.954–1.015)	0.307	
Hypertension				0.272
Yes	209/579	0.977 (0.949–1.006)	0.113	
No	166/544	0.935 (0.871–1.004)	0.063	
Diabetes				0.058
Yes	110/309	0.755 (0.577–0.986)	0.039	
No	265/814	0.981 (0.955–1.007)	0.147	
Smoking				0.751
Yes	88/230	0.977 (0.925–1.031)	0.399	
No	287/893	0.965 (0.932–0.998)	0.041	
AKI				0.723
Yes	223/512	0.978 (0.939–1.018)	0.271	
No	152/611	0.969 (0.931–1.008)	0.121	

CALLY index, CRP-albumin-lymphocyte index; AKI, Acute kidney injury.

### Association between the AKI occurrence and CALLY index

There was a statistically significant difference in the occurrence of AKI among the groups (Q1: 60.9% vs. Q2: 49.8% vs. Q3: 39.1% vs. Q4: 32.5%, *p* < 0.001) (Table 1), indicating a decreasing trend with the CALLY index (Figure 4C). Furthermore, the AUC of the CALLY index for discriminating AKI occurrence was 0.628 (95%CI: 0.596–0.661, *p* < 0.001) (Supplementary Table S4). The cut-off value for the CALLY index was determined to be 12.69 for AKI occurrence (Figure 5C). The baseline characteristics of the Non-AKI and AKI groups were presented in Supplementary Table S5. In comparison to individuals in the Non-AKI group, those in the AKI group experienced higher 30-day mortality, 60-day mortality, ICU mortality, hospital mortality, longer ICU stays, and shorter hospital stays. The CALLY index in the Non-AKI group was significantly higher than in the AKI group (21.39 vs. 11.02, *p* < 0.001). Figure 2C displayed the distribution characteristics of the CALLY index based on AKI occurrence.

Supplementary Table S6 presented the results of binary logistic regression analysis on the risk of AKI occurrence in sepsis patients, including variables from the univariate analysis. Logistic regression analysis demonstrated a significant association between the CALLY index and AKI occurrence in both the unadjusted model [HR (95%CI): 0.981 (0.965–0.997); *p* = 0.019], partially adjusted model [HR (95%CI): 0.980 (0.964–0.996); *p* = 0.012], and fully adjusted model [HR (95%CI): 0.980 (0.962–0.998); *p* = 0.033]. The risk of AKI occurrence in the Q2, Q3, and Q4 groups of the CALLY index was lower compared to the Q1 group, demonstrating a decreasing trend with increasing CALLY index values [Q1 vs. Q2: HR, 0.651 (95%CI: 0.412–1.028); Q3: HR, 0.360 (95%CI: 0.226–0.572); Q4: HR, 0.200 (95%CI: 0.123–0.326); *p* for trend < 0.001] (Figure 6C; Supplementary Table S6).

The risk stratification value of the CALLY index for AKI occurrence was further analyzed in multiple subgroups of the enrolled patients, including age, gender, hypertension, diabetes, smoking, and lactate (Supplementary Table S7). The CALLY index showed a significant association with an increased risk of AKI occurrence in several subgroups: individuals aged >65 years [HR (95%CI): 0.971 (0.949–0.992)], male [HR (95%CI): 0.930 (0.867–0.998)], those without hypertension [HR (95%CI): 0.939 (0.888–0.994)], those without diabetes [HR (95%CI): 0.975 (0.952–0.998)], those without smoking [HR (95%CI): 0.981 (0.965–0.998)], and those lactate level > 2.0 mmol/L [HR (95%CI): 0.970 (0.946–0.995)] (all *p* < 0.05).

## Discussion

To the best of our knowledge, this study was the first to investigate the relationship between the CALLY index and clinical outcomes in a critically ill population with sepsis. The findings of this study revealed that a lower CALLY index was associated with increased 30- and 60-day mortality rates, as well as an increased risk of AKI occurrence in critically ill patients with sepsis. Notably, these associations remained significant even after adjusting for various clinical and laboratory variables. These results suggested that the CALLY index could serve as a novel, simple, and efficient biomarker for clinicians, and may also act as an independent risk factor in critically ill patients with sepsis.

The CALLY index, which included CRP, serum albumin, and lymphocyte levels, is used to assess inflammation, nutrition, and immune function. These factors played significant roles in the progression of sepsis (26, 29, 32). The association between the CALLY index and clinical outcomes in sepsis patients can be examined from three perspectives: inflammation level, nutrition status, and immune function. In sepsis, an imbalance in immune responses can lead to excessive inflammatory reactions characterized by the release of inflammatory factors (38). This triggered systemic inflammation and multiple organ failure. On the other hand, immune imbalance can also result in anti-inflammatory hyperactivity and the production of anti-inflammatory cytokines, which affected immune cell functions and disrupted the body’s equilibrium. Inflammation markers were crucial indicators throughout the development of sepsis (39). CRP, a commonly used inflammatory marker in clinical practice, had been studied for its prognostic efficacy in sepsis (39). Studies had shown that elevated CRP levels in sepsis patients were associated with increased risk of in-hospital mortality rates and short-term mortality. For instance, Memiş et al. (40) found that serum C-reactive protein was a predictor of survival in patients with severe sepsis. Another study of 349 sepsis patients identified CRP as an independent predictor of short-term mortality (41). Furthermore, a prospective study of 851 sepsis patients demonstrated that an admission CRP level > 100 mg/L was associated with 30-day mortality (42).

Apart from inflammation, the impact of nutritional status on the occurrence and progression of sepsis should not be overlooked (39). During the acute phase of sepsis, severe infection triggered the release of a large amount of inflammatory mediators in the body, promoting the secretion of catabolic hormones such as epinephrine, cortisol, and glucagon. These hormones increased glucose production, glycogen breakdown, fat mobilization, as well as protein metabolism and breakdown, leading to malnutrition in patients (43–46). The immune response triggered by infection also increases basal metabolism, further exacerbating malnutrition (47). On the other hand, malnutrition can result in immune suppression, affecting the control of infections (48). Negative energy balance in septic patients was associated with an increased risk of infection, organ failure, prolonged mechanical ventilation, and extended hospital stay (20). Albumin, a protein synthesized by the liver, had been widely used as an indicator of malnutrition in the clinical setting (49). In sepsis, the underlying inflammatory state decreased albumin production in the liver by increasing the levels of inflammatory factors, which was the primary cause of hypoalbuminemia occurring early in sepsis (50). In a study conducted in 2019 with 577 consecutive severe sepsis patients, low serum albumin levels were strongly associated with poor outcomes (21). Similarly, a retrospective cohort study found that albumin was a predictor of the severity of abdominal sepsis in adult patients (51). Numerous studies had reported that serum albumin level was closely associated with inflammation and nutrition and was significantly associated with prognosis in patients with sepsis (52–54).

In addition to inflammation levels and nutritional status, immune function played a crucial role in the occurrence and development of sepsis. The initial phase of sepsis was characterized by a strong inflammatory response, which was believed to be accompanied by a downregulation of immune cell function (55). This included lymphocytes, dendritic cells, and neutrophils, leading to immunosuppression and worsening patient outcomes (56, 57). One significant aspect of immunosuppression during sepsis was the apoptosis (cell death) of immune cells, similar to the decreased levels of circulating lymphocytes. Apoptosis, autophagy, and other factors contributed to the early reduction in lymphocyte count (58–61). Previous studies had indicated that lymphocyte apoptosis played a critical role in the immunosuppressive stage of sepsis (62). This suggested that factors associated with lymphocyte apoptosis could potentially serve as prognostic predictors for sepsis patients. Several reports had shown that lymphocyte apoptosis occurred rapidly after the onset of sepsis, resulting in profound and persistent loss of lymphocytes, which was associated with poor outcomes (63, 64). Supporting this, Jiang et al. (65) found that increased lymphocyte apoptosis and decreased lymphocyte counts were both independent risk indicators for 28-day mortality. Prolonged lymphopenia was identified as the main risk factor for sepsis-induced death in older adult patients (63). A retrospective study conducted in Spain demonstrated that sepsis patients with lymphocytopenia (low lymphocyte count) had higher rates of ICU admission and mortality (66).

In combination, CRP, serum albumin, and lymphocyte levels may be more valuable and reliable biomarkers for predicting outcomes across various diseases, providing both inflammation level, nutritional status, and immune function information (33, 34). Tsunematsu et al. indicated that the CALLY index can predict long-term outcomes in patients with distal cholangiocarcinoma after pancreaticoduodenectomy, highlighting the importance of a comprehensive assessment of inflammatory status (67). Müller et al. (68) found that the CALLY index was an independent prognostic predictor for overall survival in hepatocellular carcinoma (HCC) patients undergoing transarterial chemoembolization (TACE). However, there was a paucity of literature concerning the correlation between the CALLY index and critically ill patients afflicted with infection-related diseases. In our investigation, we aimed to elucidate the utility of the CALLY index in forecasting the prognosis of sepsis patients. Our study revealed that the CALLY index emerged as a noteworthy independent risk predictor for both 30-/60-day mortality and the incidence of AKI among sepsis patients. These results suggested that early decrease of the CALLY index could potentially serve as an indicator of adverse outcomes in sepsis patients. Consequently, we proposed that enhanced monitoring of CALLY index may be more useful in guiding clinical decision-making for individuals afflicted with sepsis.

In addition, this study performed a subgroup analysis to further evaluate the risk stratification of different patient groups. The results showed that the predictive value of the CALLY index for 30-/60-day mortality and AKI occurrence remained consistent in both male and female patients. However, no significant association was found between the CALLY index and short-term mortality or AKI occurrence in patients with hypertension or diabetes who were included in the study. This phenomenon may be attributed to reverse causality, as patients with these comorbidities might have received appropriate treatment or adopted healthier lifestyle habits, thereby improving their prognosis despite their higher risk of short-term mortality. Furthermore, the study observed that patients with lower CALLY index tended to be older, and the relationship between the CALLY index and poor outcomes appeared to be more pronounced in older patients. Therefore, clinicians should be attentive to older patients who are more likely to have multiple comorbidities. However, similar attention should also be given to younger patients, as they may still face a high mortality rate despite their younger age. To summarize, the findings from this analysis indicated that the CALLY index should not be regarded as a standalone diagnostic tool but rather used in conjunction with other clinical and laboratory parameters to provide a comprehensive assessment of an individual’s metabolic health and risk stratification for developing clinical outcomes.

Our study provided confirmation that the CALLY index can serve as an effective predictor in clinical practice and was an independent risk predictor for 30-day mortality, 60-day mortality, and AKI occurrence. However, we must acknowledge several limitations. First, as a single-center retrospective study, it was subject to biases inherent in such study designs, including selection bias and information bias. Prospective studies would be needed to confirm the prognostic value of the CALLY index in real-time clinical decision-making. Second, despite utilizing multivariate adjustment and subgroup analyses, there may still be residual or unmeasured confounding factors that could have influenced the clinical outcomes, including aspects like hemodynamic monitoring, fluid resuscitation, appropriate vasoactive drug, and antibiotic therapy. Future research should aim to investigate the potential impact of these management protocols and treatments on the predictive capability of the CALLY index for clinical prognosis. Third, this study only analyzed the baseline CALLY index, and dynamic changes in the index throughout the hospital and ICU stay were not examined. Future studies should focus on exploring the changes and predictive value of the CALLY index at different time to predict sepsis outcome. Moreover, the study focused on sepsis patients aged 18 years and older from China, and the association may not be fully generalizable to the general population or disease population. Further studies are needed for validation in other populations and health conditions. Nonetheless, this study represented one of the initial investigations into the potential use of the CALLY index as a biomarker for assessing the risk of adverse outcomes in sepsis patients. Additionally, the robustness of the associations had been confirmed through various regression models.

## Conclusion

In summary, our study expanded the application of the CALLY index to critically ill patients with sepsis and showed its potential as a risk stratification tool for predicting 30-/60-day mortality and occurrence of AKI in this patient population. These findings emphasized the significance of incorporating the CALLY index into physicians’ daily practice. However, further research is necessary to investigate whether optimizing control of the CALLY index can enhance clinical prognosis in the future.

## Data availability statement

The original contributions presented in the study are included in the article/Supplementary material, further inquiries can be directed to the corresponding authors.

## Ethics statement

The studies involving humans were approved by Affiliated Hospital of Jiangsu University (No. KY2023K1007). The studies were conducted in accordance with the local legislation and institutional requirements. The participants provided their written informed consent to participate in this study.

## Author contributions

JZ: Conceptualization, Data curation, Formal Analysis, Supervision, Writing – original draft, Writing – review & editing. QZ: Conceptualization, Data curation, Writing – review & editing. SL: Conceptualization, Writing – review & editing. NY: Conceptualization, Writing – review & editing. ZH: Conceptualization, Supervision, Writing – original draft, Writing – review & editing.
