# Supplementary material for: Clinical predictive value of the CRP-albumin-lymphocyte index for prognosis of critically ill patients with sepsis in intensive care unit: a retrospective single-center observational study
Source: Front Public Health. 2024 May 22;12:1395134. doi: 10.3389/fpubh.2024.1395134 (PMC11150768; doi:10.3389/fpubh.2024.1395134)
Supplement: Supplementary file 1 [file Table_1.docx]

**Supplementary material**

Supplementary Table 1: Baseline characteristics of the Q1-Q3 and Q4 groups.

Supplementary Table 2: The diagnostic accuracy of various prediction factors for 30/60 days mortality.

Supplementary Table 3: Cox proportional hazards regression of the factors influencing all-cause death of the study population.

Supplementary Table 4: Baseline characteristics of the Non-AKI and AKI groups.

Supplementary Table 5: The diagnostic accuracy of various prediction factors for AKI occurrence.

Supplementary Table 6: Binary logistic regression analysis of the factors influencing AKI occurrence of the study population.

Supplementary Table 7: Logistic regression models for AKI occurrence.

Supplementary Table 8: Subgroup analysis regarding the influence of different CALLY index in the AKI occurrence.

Table S1. Baseline characteristics of the Q1-Q3 and Q4 groups

| **Variables** | **Overall** | **Q1-Q3 group** | **Q4 group** | **P-value** |
| --- | --- | --- | --- | --- |
| N | 1123 | 843 | 280 |  |
| Age, years | 75 (65-84) | 76 (66-84) | 75 (64-85) | 0.736 |
| Male, n (%) | 707 (63.0) | 537 (63.7) | 170 (60.7) | 0.370 |
| BMI, kg/m^2^ | 22.49 (20.08-25.21) | 22.49 (20.11-25.10) | 22.49 (19.99-25.36) | 0.658 |
| Smoking, n (%) | 229 (20.4) | 179 (21.3) | 50 (17.9) | 0.221 |
| **Comorbidities, n (%)** | | | | |
| Hypertension | 579 (51.3) | 442 (52.4) | 137 (48.9) | 0.309 |
| Diabetes | 309 (27.5) | 235 (27.9) | 74 (26.4) | 0.638 |
| Coronary artery disease | 116 (10.3) | 92 (10.9) | 24 (8.6) | 0.272 |
| COPD | 87 (7.7) | 56 (6.6) | 31 (11.1) | 0.016 |
| Cerebral infarction | 161 (14.3) | 120 (14.2) | 41 (14.6) | 0.866 |
| **Infection pathogens, n (%)** | | | | |
| Gram-positive bacteria | 136 (12.1) | 101 (12.0) | 35 (12.5) | 0.818 |
| Gram-negative bacteria | 335 (29.8) | 275 (32.6) | 60 (21.4) | <0.001 |
| Fungus | 77 (6.9) | 68 (8.1) | 9 (3.2) | 0.005 |
| Virus | 60 (5.3) | 49 (5.8) | 11 (3.9) | 0.225 |
| **Infection sites, n (%)** | | | | |
| Multisite Infection | 120 (10.7) | 110 (13.0) | 10 (3.6) | <0.001 |
| Lower respiratory infection | 436 (38.8) | 322 (38.2) | 114 (40.7) | 0.454 |
| Gastrointestinal infection | 11 (1.0) | 9 (1.1) | 2 (0.7) | 0.603 |
| Intra-abdominal infection | 392 (34.9) | 285 (33.8) | 107 (38.2) | 0.180 |
| Genitourinary tract infection | 69 (6.1) | 53 (6.3) | 16 (5.7) | 0.729 |
| Bacteremia | 10 (0.9) | 7 (0.8) | 3 (1.1) | 0.710 |
| Skin and soff tissue infection | 85 (7.6) | 57 (6.8) | 28 (10.0) | 0.076 |
| **Laboratory tests** | | | | |
| WBC *10^9^ /L | 11.4 (7.4-17.1) | 11.5 (7.4-17.3) | 11.2 (7.4-16.6) | 0.613 |
| Neu *10^9^ /L | 10.1 (6.3-15.5) | 10.3 (6.5-16.1) | 9.3 (6.1-14.6) | 0.027 |
| Lym *10^9^ /L | 0.6 (0.3-0.9) | 0.5 (0.3-0.7) | 0.9 (0.6-1.4) | <0.001 |
| Mon *10^9^ /L | 0.4 (0.2-0.7) | 0.4 (0.2-0.6) | 0.5 (0.2-0.8) | 0.001 |
| Hb, g/dL | 115 (97-130) | 112 (95-128) | 119 (103-137) | <0.001 |
| PLT *10^9^ /L | 149 (95-214) | 136 (86-201) | 189 (139-256) | <0.001 |
| CRP, mg/L | 104.2 (42.0-163.2) | 132.3 (86.5-186.5) | 12.3 (4.0-32.8) | <0.001 |
| CALLY index | 15.52 (7.16-49.58) | 10.49 (5.64-19.74) | 199.07 (89.45-653.83) | <0.001 |
| Tbil, μmol/L | 17.4 (10.9-28.2) | 18.3 (11.9-30.1) | 13.9 (8.4-23.3) | <0.001 |
| ALT, U/L | 32.0 (21.0-56.0) | 32.0 (21.0-58.0) | 32.0 (21.0-50.0) | <0.001 |
| AST, U/L | 38.1 (23.9-73.0) | 40.0 (24.3-80.0) | 33.5 (22.0-63.6) | 0.393 |
| Albumin, g/L | 28.2 (24.2-33.2) | 27.1 (23.5-31.7) | 32.2 (28.3-36.9) | 0.006 |
| Glucose, mmol/L | 8.2 (6.6-11.8) | 8.4 (6.6-12.1) | 7.88 (6.52-10.14) | <0.001 |
| Creatinine, μmol/L | 92.6 (63.7-153.1) | 109.6 (66.3-163.8) | 76.6 (56.6-123.7) | 0.023 |
| BUN, mmol/L | 8.89 (6.04-13.95) | 9.69 (6.37-14.95) | 7.17 (5.24-10.61) | <0.001 |
| Uric acid, μmol/L | 286.9 (192.3-411.7) | 291.7 (192.4-414.3) | 276.1 (190.2-400.6) | <0.001 |
| D-dimer, mg/L | 4.2 (2.1-8.4) | 4.8 (2.4-8.8) | 3.4 (1.6-7.2) | 0.242 |
| Potassium, mmol/L | 3.7 (3.3-4.2) | 3.7 (3.3-4.2) | 3.8 (3.3-4.2) | <0.001 |
| Lactate, mmol/L | 2.1 (1.4-3.6) | 2.1 (1.5-3.7) | 2.0 (1.3-3.4) | 0.110 |
| **Severity scoring** |  |  |  |  |
| APACHE II score | 25 (19-30) | 25 (20-30) | 25 (18-30) | 0.480 |
| SOFA score | 12 (10-14) | 12 (10-14) | 12 (9-14) | 0.021 |
| **Treatments** | | | | |
| CRRT, n (%) | 78 (6.9) | 65 (7.7) | 13 (4.6) | 0.080 |
| Vasoactive drug, n (%) | 748 (66.6) | 601 (71.3) | 147 (52.4) | <0.001 |
| Invasive ventilation, n (%) | 752 (67.0) | 557 (66.1) | 195 (69.6) | 0.271 |
| **Endpoints** | | | | |
| 30-day mortality, n (%) | 316 (28.1) | 270 (32.0) | 46 (16.4) | <0.001 |
| 60-day mortality, n (%) | 375 (33.4) | 317 (37.6) | 58 (20.7) | <0.001 |
| AKI, n (%) | 512 (45.6) | 421 (49.9) | 91 (32.5) | <0.001 |
| Length of ICU stay, days | 6 (3-12) | 6 (3-12) | 5 (2-11) | <0.001 |
| Length of hospital stay, days | 16 (11-25) | 16 (10-26) | 17 (11-25) | 0.406 |
| ICU mortality, n (%) | 358 (31.9) | 302 (35.8) | 56 (20.2) | <0.001 |
| Hospital mortality, n (%) | 379 (33.7) | 320 (38.0) | 59 (21.1) | <0.001 |

Abbreviations: CALLY index, CRP-albumin-lymphocyte index; BMI, body mass index; COPD, chronic obstructive pulmonary disease; WBC, white blood cell count; Neu, neutrophil; Lym, lymphocyte; Mon, monocyte; Hb, hemoglobin; PLT, platelet; CRP, C-reactive protein; Tbil, total bilirubin; ALT, alanine transaminase; AST, aspartate aminotransferase; BUN, blood urea nitroge; APACHE II, Acute Physiology and Chronic Health Evaluation II; SOFA, Sequential Organ Failure Assessment; CRRT, continuous renal replacement therapy; AKI, Acute kidney injury; ICU, Intensive Care Unit.

Table S2. The diagnostic accuracy of various prediction factors for 30/60 days mortality

| Variables | 30-day mortality | | | | | 60-day mortality | | | | |
| --- | --- | --- | --- | --- | --- | --- | --- | --- | --- | --- |
|  | AUC (95%CI) | Cut-off value | Sensitivity | Specificity | P-value | AUC (95%CI) | Cut-off value | Sensitivity | Specificity | P-value |
| CALLY index | 0.617 (0.582-0.653) | 20.20 | 0.478 | 0.709 | <0.001 | 0.615 (0.581-0.649) | 22.25 | 0.469 | 0.724 | <0.001 |
| WBC | 0.543 (0.506-0.580) | 12.6 | 0.506 | 0.582 | 0.024 | 0.545 (0.509-0.580) | 12.6 | 0.501 | 0.587 | 0.015 |
| Neu | 0.553 (0.516-0.590) | 8.2 | 0.690 | 0.403 | 0.005 | 0.558 (0.523-0.594) | 7.9 | 0.709 | 0.392 | 0.002 |
| Lym | 0.614 (0.579-0.649) | 0.8 | 0.387 | 0.794 | <0.001 | 0.609 (0.576-0.643) | 0.9 | 0.338 | 0.854 | <0.001 |
| CRP | 0.552 (0.516-0.589) | 30.9 | 0.883 | 0.238 | 0.006 | 0.547 (0.512-0.583) | 30.9 | 0.877 | 0.245 | 0.010 |
| Albumin | 0.560 (0.523-0.598) | 27.2 | 0.611 | 0.525 | 0.002 | 0.567 (0.531-0.603) | 27.2 | 0.617 | 0.526 | <0.001 |
| APACHE II score | 0.612 (0.576-0.648) | 27 | 0.504 | 0.644 | <0.001 | 0.613 (0.579-0.647) | 27 | 0.510 | 0.666 | <0.001 |
| SOFA score | 0.608 (0.572-0.644) | 13 | 0.491 | 0.638 | <0.001 | 0.612 (0.577-0.646) | 13 | 0.480 | 0.650 | <0.001 |

Abbreviations: CALLY index, CRP-albumin-lymphocyte index; WBC, white blood cell count; Neu, neutrophil; Lym, lymphocyte; CRP, C-reactive protein; APACHE II, Acute Physiology and Chronic Health Evaluation II; SOFA, Sequential Organ Failure Assessment.

Table S3. Cox proportional hazards regression of the factors influencing all-cause death of the study population.

| **Variables** | **HR** | **95% CI** | **P-value** |
| --- | --- | --- | --- |
| CALLY index | 0.540 | 0.409-0.714 | <0.001 |
| Age | 1.019 | 1.011-1.027 | <0.001 |
| Male | 0.953 | 0.770-1.180 | 0.660 |
| BMI | 0.976 | 0.953-1.000 | 0.048 |
| Smoking | 1.145 | 0.901-1.456 | 0.269 |
| Hypertension | 1.161 | 0.948-1.423 | 0.149 |
| Diabetes | 1.153 | 0.924-1.439 | 0.206 |
| WBC | 1.014 | 1.001-1.026 | 0.028 |
| Creatinine | 1.001 | 1.001-1.002 | <0.001 |
| BUN | 1.027 | 1.021-1.034 | <0.001 |
| Uric acid | 1.001 | 1.001-1.002 | <0.001 |
| D-dimer | 1.015 | 1.011-1.019 | <0.001 |
| Potassium | 1.005 | 0.979-1.031 | 0.718 |
| Lactate | 1.102 | 1.075-1.130 | <0.001 |
| APACHE II score | 1.044 | 1.032-1.057 | <0.001 |
| SOFA score | 1.078 | 1.052-1.105 | <0.001 |
| Invasive ventilation | 3.459 | 2.490-4.804 | <0.001 |

Abbreviations: CALLY index, CRP-albumin-lymphocyte index; BMI, body mass index; WBC, white blood cell count; BUN, blood urea nitroge; APACHE II, Acute Physiology and Chronic Health Evaluation II; SOFA, Sequential Organ Failure Assessment; AKI, Acute kidney injury.

Table S4. The diagnostic accuracy of various prediction factors for AKI occurrence

| Variables | AKI occurrence | | | | |
| --- | --- | --- | --- | --- | --- |
|  | AUC (95%CI) | Cut-off value | Sensitivity | Specificity | P-value |
| CALLY index | 0.628 (0.596-0.661) | 12.69 | 0.664 | 0.555 | <0.001 |
| WBC | 0.583 (0.549-0.617) | 12.6 | 0.529 | 0.63 | <0.001 |
| Neu | 0.590 (0.556-0.624) | 11.2 | 0.543 | 0.628 | <0.001 |
| Lym | 0.556 (0.522-0.590) | 0.7 | 0.455 | 0.658 | 0.001 |
| CRP | 0.627 (0.595-0.660) | 121.5 | 0.539 | 0.689 | <0.001 |
| Albumin | 0.562 (0.528-0.596) | 27.9 | 0.591 | 0.547 | <0.001 |
| APACHE II score | 0.607 (0.574-0.640) | 28 | 0.471 | 0.706 | <0.001 |
| SOFA score | 0.605 (0.572-0.638) | 13 | 0.519 | 0.650 | <0.001 |

Abbreviations: CALLY index, CRP-albumin-lymphocyte index; WBC, white blood cell count; Neu, neutrophil; Lym, lymphocyte; CRP, C-reactive protein; APACHE II, Acute Physiology and Chronic Health Evaluation II; SOFA, Sequential Organ Failure Assessment; AKI, Acute kidney injury.

Table S5. Baseline characteristics of the Non-AKI and AKI groups

| **Variables** | **Overall** | **Non-AKI group** | **AKI group** | **P-value** |
| --- | --- | --- | --- | --- |
| N | 1123 | 611 | 512 |  |
| Age, years | 75 (65-84) | 75 (63-85) | 76 (66-84) | 0.336 |
| Male, n (%) | 707 (63.0) | 390 (63.8) | 317 (61.9) | 0.508 |
| BMI, kg/m^2^ | 22.49 (20.08-25.21) | 22.49 (20.07-24.97) | 22.49 (20.20-25.40) | 0.639 |
| Smoking, n (%) | 229 (20.4) | 134 (21.9) | 95 (18.6) | 0.167 |
| **Comorbidities, n (%)** | | | | |
| Hypertension | 579 (51.3) | 301 (49.3) | 278 (54.3) | 0.093 |
| Diabetes | 309 (27.5) | 146 (23.9) | 163 (31.8) | 0.003 |
| Coronary artery disease | 116 (10.3) | 46 (7.5) | 70 (13.7) | 0.001 |
| COPD | 87 (7.7) | 51 (8.3) | 36 (7.0) | 0.411 |
| Cerebral infarction | 161 (14.3) | 86 (14.1) | 75 (14.6) | 0.785 |
| **Infection pathogens, n (%)** | | | | |
| Gram-positive bacteria | 136 (12.1) | 73 (11.9) | 63 (12.3) | 0.855 |
| Gram-negative bacteria | 335 (29.8) | 153 (25.0) | 182 (35.5) | <0.001 |
| Fungus | 77 (6.9) | 27 (4.4) | 50 (9.8) | <0.001 |
| Virus | 60 (5.3) | 42 (6.9) | 18 (3.5) | 0.013 |
| **Infection sites, n (%)** | | | | |
| Multisite Infection | 120 (10.7) | 43 (7.0) | 77 (15.0) | <0.001 |
| Lower respiratory infection | 436 (38.8) | 290 (47.5) | 146 (28.5) | <0.001 |
| Gastrointestinal infection | 11 (1.0) | 2 (0.3) | 9 (1.8) | 0.015 |
| Intra-abdominal infection | 392 (34.9) | 207 (33.9) | 185 (36.1) | 0.430 |
| Genitourinary tract infection | 69 (6.1) | 16 (2.6) | 53 (10.4) | <0.001 |
| Bacteremia | 10 (0.9) | 3 (0.5) | 7 (1.4) | 0.120 |
| Skin and soff tissue infection | 85 (7.6) | 51 (8.3) | 34 (6.6) | 0.282 |
| **Laboratory tests** | | | | |
| WBC *10^9^ /L | 11.4 (7.4-17.1) | 10.5 (7.0-15.4) | 12.9 (8.0-18.9) | <0.001 |
| Neu *10^9^ /L | 10.1 (6.3-15.5) | 9.2 (6.1-14.0) | 11.8 (6.8-17.4) | <0.001 |
| Lym *10^9^ /L | 0.6 (0.3-0.9) | 0.6 (0.4-0.9) | 0.5 (0.3-0.8) | 0.001 |
| Mon *10^9^ /L | 0.4 (0.2-0.7) | 0.4 (0.2-0.6) | 0.4 (0.2-0.7) | 0.965 |
| Hb, g/dL | 115 (97-130) | 117 (100-131) | 112 (93-129) | 0.002 |
| PLT *10^9^ /L | 149 (95-214) | 167 (115-233) | 128 (78-192) | <0.001 |
| CRP, mg/L | 104.2 (42.0-163.2) | 84.3 (31.5-149.8) | 129.3 (61.1-186.7) | <0.001 |
| CALLY index | 15.52 (7.16-49.58) | 21.39 (9.25-74.20) | 11.02 (5.44-28.39) | <0.001 |
| Tbil, μmol/L | 17.4 (10.9-28.2) | 15.8 (10.0-23.3) | 20.9 (12.2-34.4) | <0.001 |
| ALT, U/L | 32.0 (21.0-56.0) | 29.0 (20.0-49.0) | 36.1 (23.0-76.1) | <0.001 |
| AST, U/L | 38.1 (23.9-73.0) | 33.0 (22.0-57.3) | 52.0 (27.0-125.8) | <0.001 |
| Albumin, g/L | 28.2 (24.2-33.2) | 29.5 (24.9-33.5) | 27.2 (23.3-32.7) | <0.001 |
| Glucose, mmol/L | 8.2 (6.6-11.8) | 7.9 (6.4-10.7) | 8.8 (6.7-13.1) | <0.001 |
| Uric acid, μmol/L | 286.9 (192.3-411.7) | 210.3 (150.0-291.5) | 401.7 (299.6-524.7) | <0.001 |
| D-dimer, mg/L | 4.2 (2.1-8.4) | 3.4 (1.8-6.4) | 6.1 (2.9-10.8) | <0.001 |
| Potassium, mmol/L | 3.7 (3.3-4.2) | 3.6 (3.3-4.0) | 3.8 (3.3-4.5) | <0.001 |
| Lactate, mmol/L | 2.1 (1.4-3.6) | 1.8 (1.3-2.7) | 2.6 (1.8-5.0) | <0.001 |
| **Severity scoring** | | | | |
| APACHE II score | 25 (19-30) | 24 (18-29) | 27 (21-33) | <0.001 |
| SOFA score | 12 (10-14) | 11 (9-14) | 13 (11-15) | <0.001 |
| **Treatments** | | | | |
| CRRT, n (%) | 78 (6.9) | 3 (0.5) | 75 (14.6) | <0.001 |
| Vasoactive drug, n (%) | 748 (66.6) | 342 (56.0) | 406 (79.3) | <0.001 |
| Invasive ventilation, n (%) | 752 (67.0) | 413 (67.6) | 339 (66.2) | 0.624 |
| **Endpoints** | | | | |
| 30-day mortality, n (%) | 316 (28.1) | 121 (19.8) | 195 (38.1) | <0.001 |
| 60-day mortality, n (%) | 375 (33.4) | 152 (24.9) | 223 (43.6) | <0.001 |
| Length of ICU stay, days | 6 (3-12) | 5 (3-11) | 7 (4-12) | <0.001 |
| Length of hospital stay, days | 16 (11-25) | 17 (11-26) | 16 (9-25) | 0.014 |
| ICU mortality, n (%) | 358 (31.9) | 146 (23.9) | 212 (41.4) | <0.001 |
| Hospital mortality, n (%) | 379 (33.7) | 156 (25.5) | 223 (43.6) | <0.001 |

Abbreviations: CALLY index, CRP-albumin-lymphocyte index; BMI, body mass index; COPD, chronic obstructive pulmonary disease; WBC, white blood cell count; Neu, neutrophil; Lym, lymphocyte; Mon, monocyte; Hb, hemoglobin; PLT, platelet; CRP, C-reactive protein; Tbil, total bilirubin; ALT, alanine transaminase; AST, aspartate aminotransferase; BUN, blood urea nitroge; APACHE II, Acute Physiology and Chronic Health Evaluation II; SOFA, Sequential Organ Failure Assessment; CRRT, continuous renal replacement therapy; AKI, Acute kidney injury; ICU, Intensive Care Unit.

Table S6. Binary logistic regression analysis of the factors influencing AKI occurrence of the study population.

| **Variables** | **OR** | **95% CI** | **P-value** |
| --- | --- | --- | --- |
| CALLY index | 0.981 | 0.965-0.997 | 0.019 |
| Age | 1.004 | 0.996-1.012 | 0.316 |
| Male | 0.921 | 0.722-1.175 | 0.508 |
| BMI | 1.013 | 0.985-1.042 | 0.352 |
| Smoking | 0.813 | 0.606-1.091 | 0.167 |
| Hypertension | 1.224 | 0.967-1.548 | 0.093 |
| Diabetes | 1.488 | 1.144-1.935 | 0.003 |
| WBC | 1.044 | 1.027-1.061 | <0.001 |
| Uric acid | 1.012 | 1.010-1.013 | <0.001 |
| D-dimer | 1.049 | 1.032-1.065 | <0.001 |
| Potassium | 1.733 | 1.473-2.040 | <0.001 |
| Lactate | 1.305 | 1.227-1.388 | <0.001 |
| APACHE II score | 1.050 | 1.034-1.066 | <0.001 |
| SOFA score | 1.104 | 1.066-1.143 | <0.001 |
| Invasive ventilation | 0.939 | 0.732-1.206 | 0.624 |

Abbreviations: CALLY index, CRP-albumin-lymphocyte index; BMI, body mass index; WBC, white blood cell count;APACHE II, Acute Physiology and Chronic Health Evaluation II; SOFA, Sequential Organ Failure Assessment; AKI, Acute kidney injury.

Table S7. Logistic regression models for AKI occurrence

| **Variables** | **Model 1** | | | **Model 2** | | | **Model 3** | | |
| --- | --- | --- | --- | --- | --- | --- | --- | --- | --- |
|  | **OR (95% CI)** | **P-value** | **P for trend** | **OR (95% CI)** | **P-value** | **P for trend** | **OR (95% CI)** | **P-value** | **P for trend** |
| **AKI occurrence** | | | | | | | | | |
| Continuous variable per unit | 0.981 (0.965-0.997) | 0.019 |  | 0.980 (0.964-0.996) | 0.012 |  | 0.980 (0.962-0.998) | 0.033 |  |
| Quartile^a^ |  |  | <0.001 |  |  | <0.001 |  |  | <0.001 |
| Q1 group |  |  |  | Ref |  |  | Ref |  |  |
| Q2 group | 0.639 (0.457-0.893) | 0.009 |  | 0.585 (0.411-0.834) | 0.003 |  | 0.651 (0.412-1.028) | 0.066 |  |
| Q3 group | 0.414 (0.295-0.581) | <0.001 |  | 0.398 (0.279-0.569) | <0.001 |  | 0.360 (0.226-0.572) | <0.001 |  |
| Q4 group | 0.310 (0.219-0.438) | <0.001 |  | 0.291 (0.202-0.420) | <0.001 |  | 0.200 (0.123-0.326) | <0.001 |  |

Model 1: unadjusted

Model 2: adjusted for age, gender, BMI, smoking, hypertension, diabetes, WBC, APACHE II score and SOFA score.

Model 3: adjusted for age, gender, BMI, smoking, hypertension, diabetes, WBC, uric acid, D-dimer, potassium, lactate, APACHE II score, SOFA score, and invasive ventilation.

a CALLY index: Q1 group (CALLY<=7.16); Q2 group (7.16<CALLY<=15.52); Q3 group (15.52<CALLY<=49.58); Q4 group (CALLY>49.58).

Abbreviations: CALLY index, CRP-albumin-lymphocyte index; BMI, body mass index; WBC, white blood cell count;APACHE II, Acute Physiology and Chronic Health Evaluation II; SOFA, Sequential Organ Failure Assessment; AKI, Acute kidney injury.

Table S8. Subgroup analysis regarding the influence of different CALLY index in the AKI occurrence.

| **Subgroups** | **No.AKI/No.patients** | **HR (95% CI)** | **P-value** | **P for interaction** |
| --- | --- | --- | --- | --- |
| Age |  |  |  | 0.082 |
| >65 | 397/840 | 0.971 (0.949-0.992) | 0.008 |  |
| <=65 | 115/283 | 0.999 (0.976-1.023) | 0.923 |  |
| Gender |  |  |  | 0.527 |
| Male | 317/707 | 0.985 (0.965-1.004) | 0.127 |  |
| Female | 195/416 | 0.974 (0.946-1.002) | 0.066 |  |
| Hypertension |  |  |  | 0.088 |
| Yes | 278/579 | 0.989 (0.972-1.005) | 0.178 |  |
| No | 234/544 | 0.939 (0.888-0.994) | 0.029 |  |
| Diabetes |  |  |  | 0.478 |
| Yes | 163/309 | 0.986 (0.964-1.009) | 0.228 |  |
| No | 349/814 | 0.975 (0.952-0.998) | 0.033 |  |
| Smoking |  |  |  | 0.784 |
| Yes | 96/230 | 0.974 (0.927-1.023) | 0.299 |  |
| No | 416/893 | 0.981 (0.965-0.998) | 0.031 |  |
| Lactate |  |  |  | 0.152 |
| >2.0 | 355/616 | 0.970 (0.946-0.995) | 0.017 |  |
| <=2.0 | 157/507 | 0.993 (0.973-1.014) | 0.509 |  |

Abbreviations: CALLY index, CRP-albumin-lymphocyte index; AKI, Acute kidney injury.
